# Significant Effects of Antiretroviral Therapy on Global Gene Expression in Brain Tissues of Patients with HIV-1-Associated Neurocognitive Disorders

**DOI:** 10.1371/journal.ppat.1002213

**Published:** 2011-09-01

**Authors:** Alejandra Borjabad, Susan Morgello, Wei Chao, Seon-Young Kim, Andrew I. Brooks, Jacinta Murray, Mary Jane Potash, David J. Volsky

**Affiliations:** 1 Molecular Virology Division, St. Luke's-Roosevelt Hospital Center and Columbia University, New York, New York, United States of America; 2 Department of Pathology and Neuroscience, The Mount Sinai Medical Center, New York, New York, United States of America; 3 Human Genomics Laboratory, Korea Research Institute of Bioscience and Biotechnology (KRIBB), Daejeon, Korea; 4 Department of Genetics, Environmental and Occupational Health Science Institute (EOHSI), Rutgers University, Piscataway, New Jersey, United States of America; Harvard University, United States of America

## Abstract

Antiretroviral therapy (ART) has reduced morbidity and mortality in HIV-1 infection; however HIV-1-associated neurocognitive disorders (HAND) persist despite treatment. The reasons for the limited efficacy of ART in the brain are unknown. Here we used functional genomics to determine ART effectiveness in the brain and to identify molecular signatures of HAND under ART. We performed genome-wide microarray analysis using Affymetrix U133 Plus 2.0 Arrays, real-time PCR, and immunohistochemistry in brain tissues from seven treated and eight untreated HAND patients and six uninfected controls. We also determined brain virus burdens by real-time PCR. Treated and untreated HAND brains had distinct gene expression profiles with ART transcriptomes clustering with HIV-1-negative controls. The molecular disease profile of untreated HAND showed dysregulated expression of 1470 genes at p<0.05, with activation of antiviral and immune responses and suppression of synaptic transmission and neurogenesis. The overall brain transcriptome changes in these patients were independent of histological manifestation of HIV-1 encephalitis and brain virus burdens. Depending on treatment compliance, brain transcriptomes from patients on ART had 83% to 93% fewer dysregulated genes and significantly lower dysregulation of biological pathways compared to untreated patients, with particular improvement indicated for nervous system functions. However a core of about 100 genes remained similarly dysregulated in both treated and untreated patient brain tissues. These genes participate in adaptive immune responses, and in interferon, cell cycle, and myelin pathways. Fluctuations of cellular gene expression in the brain correlated in Pearson's formula analysis with plasma but not brain virus burden. Our results define for the first time an aberrant genome-wide brain transcriptome of untreated HAND and they suggest that antiretroviral treatment can be broadly effective in reducing pathophysiological changes in the brain associated with HAND. Aberrantly expressed transcripts common to untreated and treated HAND may contribute to neurocognitive changes defying ART.

## Author Summary

HAND is a common complication of HIV-1 infection in the nervous system presenting a varied spectrum of clinical manifestations with cognitive, motor and behavioral symptoms. Introduction of ART has greatly reduced morbidity and mortality in HIV-1 infection; however HAND persists and its overall prevalence appears to have increased despite treatment. The effects of the treatment on neurological disease are not well understood. Here, we used genomic analysis to compare gene expression profiles in brain tissues from treated and untreated patients who died with HAND. We identified a large number of genes and biological pathways dysregulated in untreated HAND compared with uninfected controls. ART appears to be effective in mitigating aberrant gene expression in brain tissues of patients with HAND but a fraction of genes remained dysregulated under ART and the patients continued to manifest HAND in the last evaluation prior to death. Our study provides new insights into the molecular changes in brain tissues of patients with HAND and the effect of the treatment on brain transcriptome. The identification of aberrantly expressed genes common to untreated and treated HAND may contribute to understand the neurocognitive impairment observed in patients under ART.

## Introduction

HAND is a common complication of HIV-1 infection in the nervous system presenting a varied spectrum of clinical manifestations with cognitive, motor and behavioral symptoms. Currently three conditions of increasing severity are recognized as components of HAND: HIV-1-associated asymptomatic neurocognitive impairment, HIV-1-associated mild neurocognitive disorders (MND), and HIV-1-associated dementia (HIV-D or HAD) [1]. HAND remains prevalent in populations with access to highly active ART, despite the efficacy of these therapies in controlling viral load and ameliorating viral load-associated clinical and neuroradiologic abnormalities [2]–[6]. By some accounts, up to 50% of HIV-1-infected individuals will develop some form of HAND regardless of access to currently available ART [7]–[9]. Under ART, HAND has became milder, its course more protracted and variable in symptoms, and it now overlaps with aging processes and potentially with other neurodegenerative diseases in AIDS patients [7], [10], [11]. The etiology of persistent cognitive deficits in patients on ART remains unclear. Studies of cerebrospinal fluid from individuals with HAND have sometimes supported contradictory conclusions; in the absence of viral replication, some authors observe associations among dementia, abnormal neurometabolites, and inflammatory phenotypes; others, with non-inflammatory states or markers of neurodegeneration [12]–[16]. There have been limited studies of brain tissues from treated patients with HAND to evaluate what biologic pathways remain abnormally regulated under ART, with studies largely focused on single cell types or molecules [17]. Comprehensive analysis of the spectrum of molecular abnormalities in the brain that may underlie HAND in the presence of ART has not yet been undertaken.

Here we used functional genomics to conduct comparative analysis of genome-wide gene expression profiles in brain tissues from treated and untreated patients who died with HAND. Functional genomics has been applied with success to identification of complex molecular pathways in carcinogenesis and to better detection, classification, and prognosis of some cancers [18]–[23]. This approach has also been used extensively to investigate the transcriptome correlates of HIV-1 infection in peripheral tissues from HIV-1-infected patients including lymph nodes [24]–[26], CD4^+^ T cells [27], [28], monocytes [29], B cells [30], and gastrointestinal mucosa [31]. Some of these studies determined the effects of ART on HIV-1 transcriptomes in patients, revealing categories of treatment-responsive genes as well as aberrantly expressed transcripts that may serve as targets for future therapies [24], [27]–[31]. In a related approach, a recent study evaluated gene expression profiles of blood monocytes as a function of ART and neuropsychological impairment of HIV-1-infected patients [32]. Interestingly in this case, there was no correlation between changes in blood monocyte transcriptomes under treatment and clinical HAND [32]. Generally, this research benefited from the ability to serially sample peripheral tissues and individual cell types in living individuals, allowing ongoing evaluation of treatment.

In contrast, analyses of human brain tissues by functional genomics can only be conducted retrospectively in autopsy tissues and are complicated by the multicellular interactions underlying central nervous system diseases [33]. Nonetheless, large-scale, cross-sectional gene expression profiling of brain tissues has revealed potential pathogenic pathways in Alzheimer's disease (AD) [34], [35], Parkinson's disease [36], [37], chronic schizophrenia [38], multiple sclerosis [39], and viral encephalitis [40]. With respect to HIV-1 infection in the brain, investigators reported transcriptional changes in selected gene categories such as anion channels in the frontal cortex of patients who died with HAND [41], [42]. Another group reported aberrant expression of genes specific to HIV-1 encephalitis (HIVE) [43], established a correlation between use of methamphetamine and up-regulation of interferon genes in these patients [44], and investigated the role of microRNA in gene regulation in HIV-1-infected brain [45]. To our knowledge, these studies did not consider the effects of ART on brain gene dysregulation in HAND.

The Manhattan HIV Brain Bank (MHBB; member of the National NeuroAIDS Tissue Consortium) follows a cohort of advanced-stage, HIV-1-infected individuals with a high prevalence of well-characterized cognitive dysfunction; with entry to the study, participants agree to be organ donors upon death. The antiviral treatment status of study participants is monitored while in the program. Thus, brain tissues obtained by this program provide an opportunity to examine the potentially diverse processes underlying HAND in the ART era. We report herein the gene expression profiles of individuals with HAND focusing on the impact of ART on these profiles.

## Results

### Effect of therapy on HIV-1 brain burdens

We assayed virus burden and cellular gene expression on archived brain tissues from 15 HIV-1-infected patients with HAND and six HIV-1-negative subjects with no neurological or neuropathological abnormalities. Information for this study group is summarized in Table 1 and Methods. All assays were performed on parallel samples from deep white matter within the anterior frontal lobe, an area implicated in HAND and HIV-1-associated neuropathologies [46], [47]. Custom consensus primers based on sequences of HIV-1 amplified from the brain were used for reliable measurement of HIV-1 in the brain by quantitative real-time PCR (QPCR) (see Methods); the results were confirmed by standard PCR and hybridization with a specific probe (Table 1 and Supplementary Figure S1). Pre-mortem plasma HIV-1 burdens are plotted for comparison (Supplementary Figure S1). With the exception of patient 30015, the HIV-1 brain burdens in untreated patients correlated with presentation of HIVE; on average, these patients had about 180-fold more viral RNA per µg total RNA than patients without HIVE. Patient 30015 had limited HIVE pathology and no detectable virus in the brain (Table 1). Patients on ART, both with and without HIVE, had lower virus burdens in the brain than untreated patients, results consistent with a previous study in a different cohort [48]. In patients with HIVE the reduction was 50% and 95% at the DNA and RNA levels respectively, while virus was undetectable in treated patients without HIVE. Notably, there was no correlation between brain and plasma viral loads and brain virus burdens were independent of patient's age, gender, ethnic background, or postmortem interval.

**Table 1:** Study subject information and HIV burdens in different compartments.

												Brain vl			
Pid	Code	Category	ART	PMI	Age	Sex	R	Risk	CD4	Plasmavl	CSFvl	DNA (copies)	RNA (copies)	Brain Pathology	ART adherence
mhbb531	C1	HIV neg	none	22	44	M	W		na		na	un	un	normal	
mhbb551	C2	HIV neg	none	24	30	F	H		na		na	un	un	normal	
mhbb567	C3	HIV neg	none	19	63	M	H		na		na	un	un	normal	
mhbb601	C4	HIV neg	none	16.5	58	F	H		na		na	un	un	normal	
mhbb594	C5	HIV neg	none	24	57	F	W		na		na	un	un	normal	
mhbb588	C6	HIV neg	none	18.5	21	M	H		na		na	un	un	normal	
mhbb500	HAND 1	HAND	none	48	47	M	W	Hom-sx	20	210000	na	115468	689556	HIVE	
mhbb537	HAND 2	HAND	none	27	45	F	B	IVDU	6	na	na	58001	902832	HIVE	
10017	HAND 3	HAND	none	4	44	M	W	IVDU	7	389120	>750000	234372	762888	HIVE	
10070	HAND 4	HAND	none	4	58	M	B	Het-sx	1	750000	>750000	471248	10689754	HIVE	
30015	HAND 5	HAND	none	6	43	M	B	Hom-sx	10	48520	134	un	un	HIVE*	
10119	HAND 6	HAND	none	6	33	M	B	Hom-sx	1	312240	<50	l.pos	un	minimal	
30013	HAND 7	HAND	none	6	30	M	B	Het-sx	8	104300	<50	l.pos	62826	ischemia	
10011	HAND 8	HAND	none	8.5	44	M	H	Hom-sx	16	162642	na	un	702	normal	
mhbb509	ART1	HAND	d4t,nor,cri	12	46	M	W	Hom-sx	203	80000	na	262933	2516	HIVE	na
10133	ART2	HAND	d4t,kal,nev	7.5	48	M	W	Hom-sx	3	173921	na	64511	448390	HIVE	80
10103	ART3	HAND	tri,kal,ten	6	40	M	H	Het-sx	15	750000	>750000	3789	854	HIVE	50
10063	ART4	HAND	d4t,aba,ten	5	51	M	H	IVDU	136	65	<50	un	un	minimal	90
10001	ART5	HAND	d4t,3tc,kal	4.5	64	F	B	IVDU	72	359	<50	un	un	minimal	60
10015	ART6	HAND	d4t,3tc,efa	20	33	M	W	Hom-sx	66	176800	na	un	un	minimal	na
20024	ART7	HAND	d4t,3tc,aba	4	62	M	W	IVDU	20	un	501	un	un	normal	95

Brain tissues and study subject information were obtained from the Manhattan HIV Brain Bank (MHBB) as described in Methods. Pid: subject I.D. at MHBB. Category abbreviation: HAND: HIV-Associated Neurocognitive Disorder. ART: antiretroviral treatment. ART drug abbreviations: nor: norvir; cri: crixivan; kal: kaletra; nev: nevirapine; tri: trizivir; ten: tenofovir; aba: abacavir; efa: efavirenz; 3tc: epivir, lamivudine; d4t: stavudine. The duration of the listed ART regimen prior to death while in the MHBB program was (in months): ART1 (5), ART2 (4), ART3 (6), ART4 (36), ART5 (24), ART6 (36), ART7 (3). PMI: post-mortem interval. R: race (W: white; H: hispanic; B: black); Risk: IVDU: intravenous drug use; Het-sx: heterosexual; Hom-sx: homosexual. CD4: number of CD4 positive T cells per mm^3^ and vl, virus loads in plasma and CSF in RNA copies per mL were provided by MHBB with other patient information. Brain vl was determined in this work by real-time PCR and expressed as viral DNA copies per 500,000 cells, the latter determined by beta-globin gene content, and viral RNA copies per 1 µg RNA; virus detection was confirmed by standard PCR and Southern hybridization with a specific probe; un: undetectable by QPCR or standard PCR; l.pos: low-positive by standard PCR; na: data not available. Brain pathology: see Methods for detailed definitions.

### Untreated HAND patients with dementia have similar brain tissue transcriptomes irrespective of their HIVE histopathology and virus burdens

**Figure 1:**
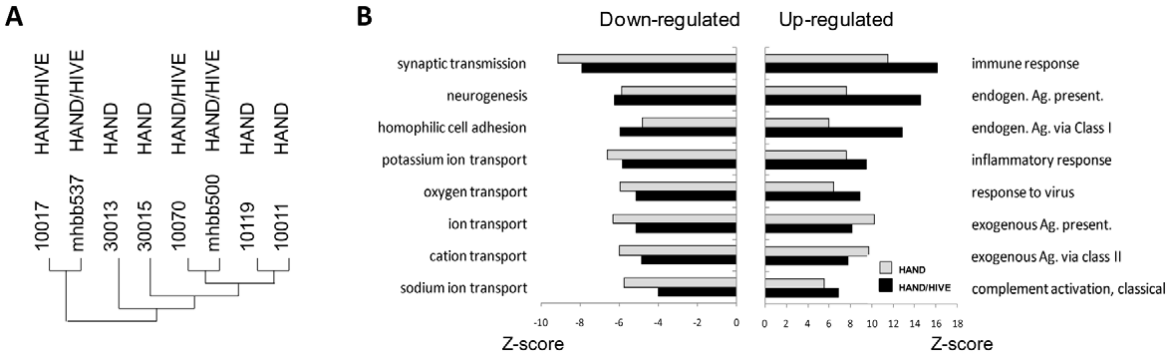
Similarity of brain transcriptomes of untreated HAND patients with and without encephalitis. **A.** Using Affymetrix microarrays we evaluated gene expression in brain tissue. Normalized data from HAND patients with or without encephalitis were analyzed by Hierarchical Clustering using Genesis software. **B.** Gene set analysis was conducted by GAzer software to identify biological pathways most significantly up-regulated (right panel) or down-regulated (left panel) in untreated HAND without or with encephalitis (HAND/HIVE) vs. uninfected controls.

Global gene expression profiles of patient brain tissues were determined on Affymetrix GeneChip Array Human Genome U133 Plus 2.0 Arrays. Three independent array experiments were performed, each comprising a subset of HIV-1-positive samples and controls, with most samples tested either in duplicates or repeated in independent runs and some samples tested three times. Replicate gene sets of the same samples were averaged, yielding 21 final brain tissue datasets for 21 subjects. Preliminary hierarchical cluster analysis of complete datasets from HAND patients indicated that the primary biological variable in clustering of these datasets was whether or not patients were on ART at the time of death (not shown). Interestingly, this analysis also indicated that brain transcriptomes from untreated patients with HIVE did not cluster independently of non-HIVE transcriptomes (Figure 1A), suggesting that they are statistically similar across their entire datasets. This result was surprising because untreated patients with HIVE had on average higher brain virus burdens than patients without HIVE (Table 1 and Supplementary Figure S1) and HIV-1 infection is known to induce cellular gene expression [49]. To confirm findings of cluster analysis we performed global gene set analysis using GAzer software [50] to identify biological pathways that were most significantly altered in HIVE positive and negative groups compared to uninfected controls (Figure 1B). GAzer employs parametric analysis of sets of co-regulated genes across complete microarray datasets, independent of arbitrary fold change (FC) value limits, thus increasing statistical power of detection of biological differences between datasets [50], [51]. Figure 1B depicts the eight most altered pathways in HAND and HAND/HIVE datasets versus controls and Supplementary Table S1 lists all aberrant pathways and detailed statistics of GAzer analysis including Z-score, p-and q-values, and Bonferroni correction value. Consistent with previous array studies in HIVE patients [43], [44], datasets of patients with HIVE showed greater dysregulation of immune responses and endogenous antigen presentation pathways than those without HIVE (Figure 1B), possibly reflecting effects of high virus burdens in the brain in untreated HIVE (Table 1). However, the two HAND patient groups were generally similar with respect to the ranking and extent of change (indicated by all four statistical measures) of the majority of altered gene sets (Figure 1B and Supplementary Table S1). Because of their overall similarities in both cluster and gene set analyses, we chose to pool microarray datasets from HAND patients with and without encephalitis for analysis of antiviral drug effects.

### Patients with HAND on antiretroviral therapy have many fewer and milder gene expression changes in the brain than untreated patients

**Figure 2:**
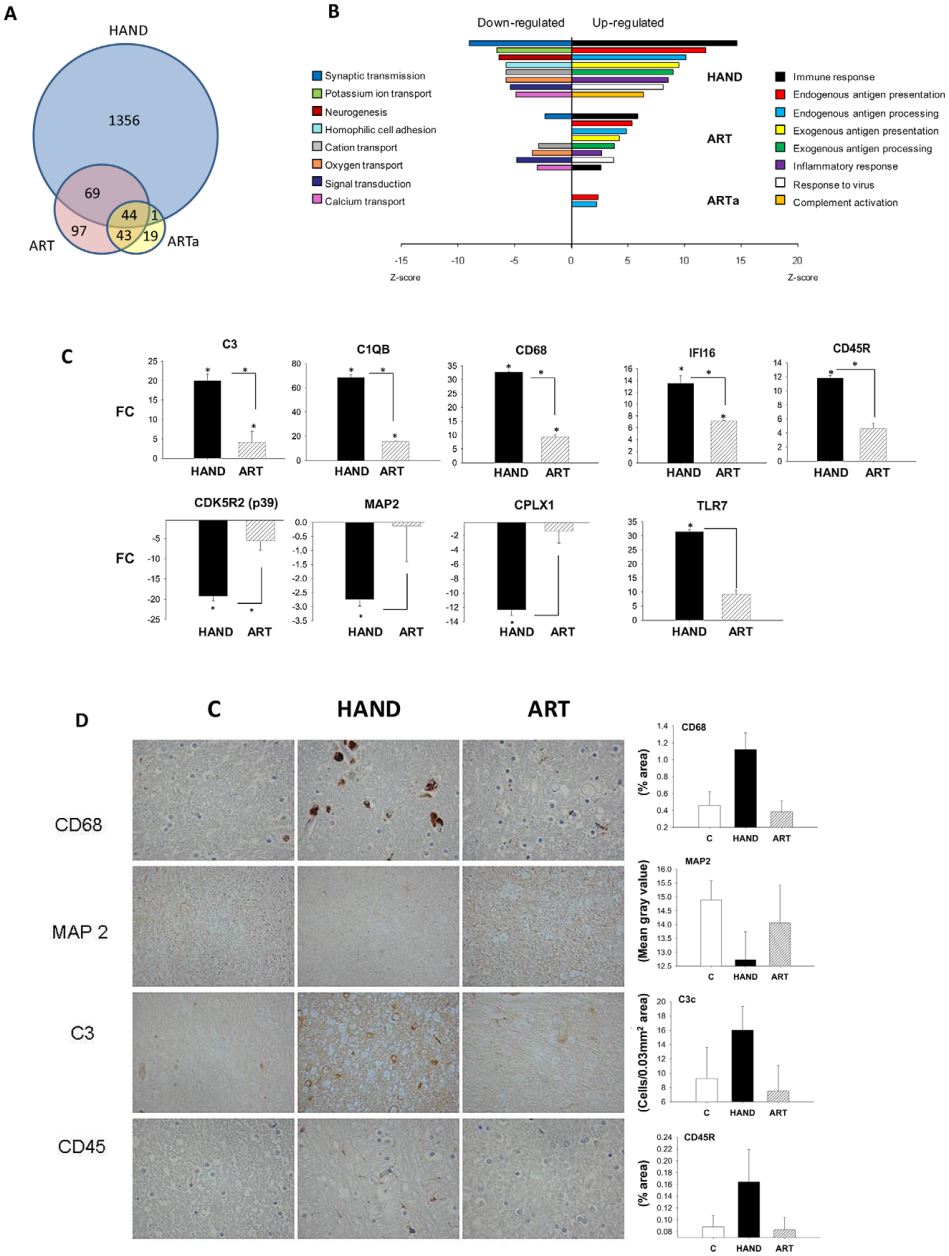
The pattern of changes in brain cell gene expression relative to uninfected subjects differs significantly between untreated HAND patients and HAND patients treated with ART. **A.** The Venn diagram displays the number and overlap of transcripts differentially expressed relative to uninfected subjects from HAND, ART, and HAND patients excluding low adherence patients ART3 and ART5 (ARTa). **B.** GAzer was employed to identify the eight most dysregulated biological pathways relative to uninfected subjects in the HAND dataset and the extent of change in the same pathways in ART and ARTa datasets are shown for comparison with Z-scores plotted for the significantly up-regulated (right panel) and down-regulated (left panel) pathways. **C.** QPCR analysis of gene expression in HAND and ARTa patients plotting fold change of expression uninfected subjects. Asterisks represent p-value<0.05 in t-test analysis, asterisks over column indicate comparison to values from uninfected subjects, asterisks between columns represent their comparison. **D.** Immunostaining and quantitation of selected proteins in brain tissue of uninfected subjects (C), HAND and ART patients.

Seven of the 15 patients in our cohort were treated using different antiretroviral drug combinations (Table 1) enabling investigation of the extent of HIV-1-induced changes in the brain and potential differences between treated and untreated patients at the level of brain transcriptomes. Patient microarray datasets were pooled separately into untreated (HAND, n = 8) and treated (ART, n = 7) groups and each group was compared to the uninfected control pool C (n = 6). We used a cut-off of 1.5 FC and p-value<0.05 to identify significantly dysregulated genes. For some analyses, we also established a subgroup ARTa excluding low adherence subjects ART3 and ART5. The complete lists of FC values and accompanying statistics for HAND, ART, and ARTa are provided in Supplementary Tables S2 and S3. Overall, we identified 2073 dysregulated transcripts in the untreated HAND group and 333 and 145 transcripts in the ART and ARTa groups, respectively (Table S2). The Venn diagram in Figure 2A depicts the number of genes dysregulated in brain tissues of untreated and treated patients with HAND and the overlap in the dysregulated genes among the three groups tested. Excluding multiple probes for the same gene and transcripts of undefined function at the time of this writing, the HAND group had 1470 genes with significantly altered expression compared to 260 in ART and 107 in ARTa (Figure 2A, Supplementary Tables S2 and S3). About two-thirds of dysregulated genes (947) in untreated patients were up-regulated and 95 of these had FC values of ≥3.0 and p = 10^−2^–10^−7^, among down-regulated genes, 58 had FC of ≤−3.0 and p of 10^−2^–10^−4^, suggesting that HIV-1 infection profoundly alters the brain transcriptome in untreated patients with HAND and indicating significant conformity of molecular profiles of disease in this cohort. In treated patients, down-modulated genes predominated, the FC values ranged from 3.95 to 1.5 for up-regulated genes and from −1.5 to −2.87 for down-regulated genes, and p-values were 5×10^−2^–7.3×10^−5^ (Supplementary Tables S2 and S3), indicating less uniformity of brain gene expression in this group. These results indicate marked differences between aberrant brain transcriptome profiles of untreated and treated patients, the latter showing 6–14-fold (depending on treatment compliance) fewer dysregulated genes with generally lesser dysregulation of expression than in untreated patients.

To confirm this observation we conducted gene ontology analysis using GAzer to examine cellular processes affected by HIV-1 infection in the brain in our patient groups. Altered gene sets (biological pathways) were identified by comparing each patient group to HIV-1-negative controls; Supplementary Table S4 lists these pathways for HAND, ART, and ARTa in the order of their significance as determined by the Z-score, p and q values, and Bonferroni statistics [50], [51]. Figure 2B shows the eight most dysregulated biological pathways in the HAND datasets, displaying the extent of dysregulation in the same pathways in ART and ARTa datasets. Up-regulated pathways in untreated HAND patients included immune responses, inflammation, response to virus, and complement activation while synaptic transmission, neurogenesis, ion transport, cell adhesion, and signal transduction were down-regulated. The statistical significance for the eight major pathway changes reached Z-scores of 6–14 and p, q and Bonferroni values of ≥10^−8^ (Figure 2B and Supplementary Table S4). In contrast, samples from treated patients showed either fewer significantly altered pathways (ART and ARTa panels in Figure 2B) or lesser extent of dysregulation of the remaining pathways displayed (*e.g.*, the ART panel in Figure 2B). The extent and kind of changes in gene ontology processes in the treatment compliant ARTa group were limited compared to changes seen in untreated patients: of the 8 most up-regulated HAND pathways only endogenous antigen presentation and processing were also up-regulated in ARTa with Z-scores less than 2.5, low significance compared to other pathways (p = 0.018 and 0.026, respectively), and no down-regulated gene sets in these categories were identified (Figure 2B and Supplementary Table S4). Notably, some pathways that were significantly down-regulated in HAND were significantly up-regulated in ARTa including neurotransmitter secretion (Z = 4.26; p = 2×10^−5^) and synaptic transmission (Z = 2.9; p = 0.0037) (Supplementary Table S4).

Dysregulation of selected genes detected by microarrays was confirmed in adjacent tissues by QPCR. Genes were chosen by previous demonstration of their link to HAND [52]–[56]. Representative gene expression values are shown in Figure 2C and the complete list is provided in Supplementary Table S5. Consistent with microarray data, brain tissues from untreated patients showed significant up-regulation of *complement component 3* (C3), *macrophage antigen CD68* (CD68), and *protein-tyrosine phosphatase receptor-type C* PTPRC (also known as CD45R); and down-regulation of *neuronal cyclin-dependent kinase 5*, *regulatory subunit 2* (CDK5R2) (only significant for p39), neuronal marker *microtubule-associate protein 2* (MAP2) and the SNARE protein *complexin 1* (CPLX1), compared to brains of patients on ART. As also noted in other studies [57], confirmatory QPCR analyses generally yielded higher FC results than parallel microarray analyses (Supplementary Table S5). Four of the changes in gene expression in the brain were also tested at the protein level by immunohistochemistry (Figure 2D). We observed increased expression of CD68, C3c and CD45R proteins in untreated HAND compared to uninfected control tissue, and an amelioration of the protein dysregulation in patients with HAND on ART. MAP2 protein was down-regulated in HAND brains compared with brains from uninfected subjects, and it was partially restored in patients under treatment for HIV-1 infection. Taken together, our findings parallel recent reports of the effects of ART on gene expression in peripheral tissues [24], [27], [29] in that treatment of HIV-1 infection by ART is also accompanied by marked reduction of the virally-induced dysregulation of gene expression in brain tissues. Treatment adherence further reduces the number of genes and biological pathways affected.

### Gene expression profiles from treated HAND patients cluster with profiles from HIV-1-negative controls

**Figure 3:**
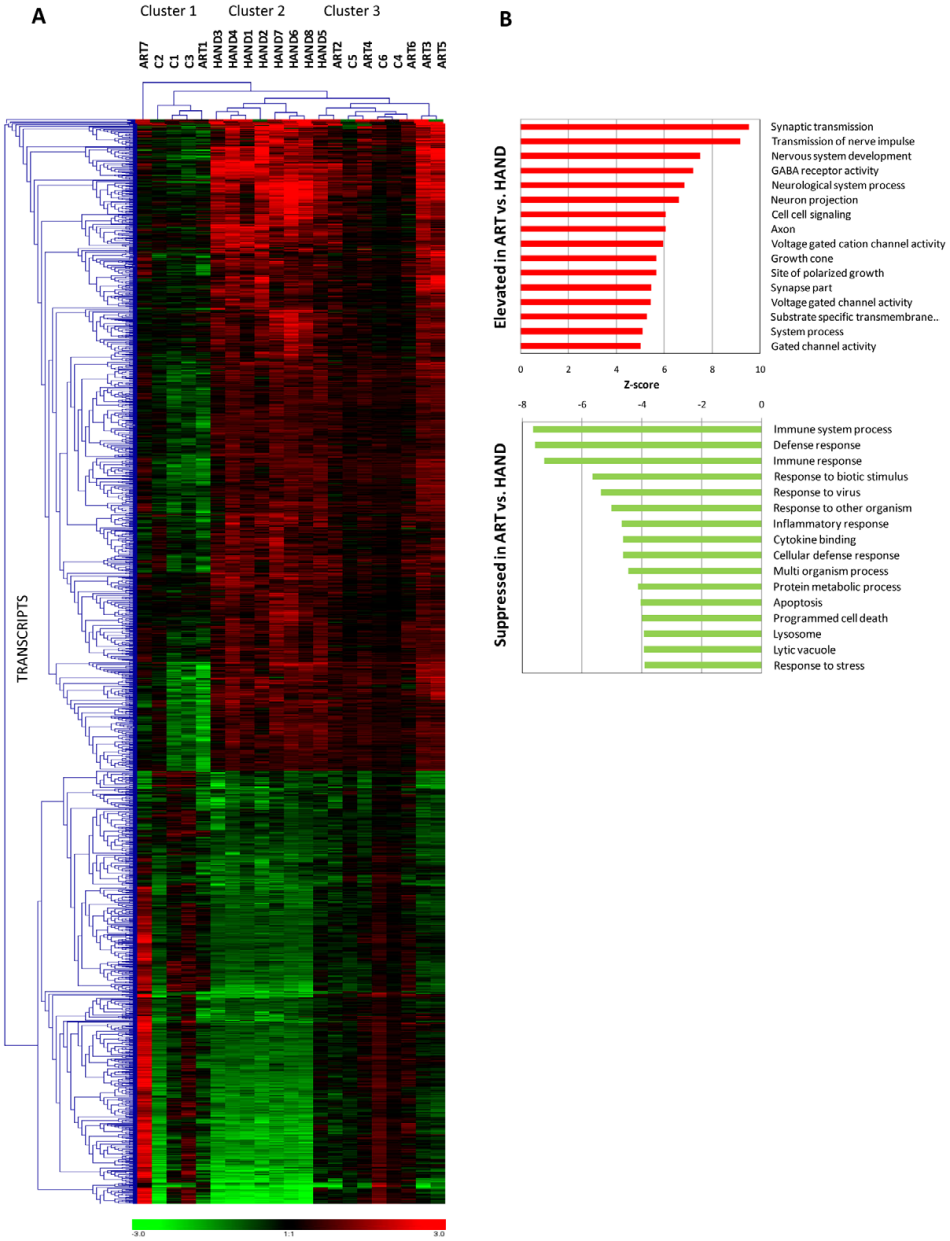
Extent and kind of differences in brain cell gene expression in untreated HAND patients and HAND patients receiving ART. **A.** Selecting the transcripts differentially expressed in brain tissue from HAND patients relative to uninfected subjects, hierarchical cluster analysis was performed on extent of expression from all subjects using Genesis software. Red represents up-regulation, green represents down-regulation. **B.** Gene ontology analysis of ART dataset vs. HAND dataset was performed using GAzer software. The upper panel shows pathways elevated in ART and the lower panel shows pathways suppressed in ART relative to HAND brain samples.

In the next level of analysis, we conducted unsupervised hierarchical clustering for 2073 dysregulated transcripts implicated in HAND (Figure 2A and Supplementary Table S2) to visualize and group individual gene expression profiles from all 21 subjects in this study (Figure 3A). We used normalized Robust Microarray Average (RMA) values from duplicate samples for each subject for greater statistical power (Methods). Three main clusters of subjects were identified. With the exception of HAND5, untreated HAND patients clustered as one group (cluster 2 in Figure 3A) distinct from the other two clusters. ART-treated HAND patients clustered with uninfected controls in two co-mingled clusters, cluster 1 containing subjects C1, C2, C3 and ART1 and cluster 3 including subjects C4, C5, C6, ART2, ART3, ART4, ART5, ART6, and the remaining untreated HAND5. Compared to cluster 3, cluster 1 was more phylogenetically distant from untreated HAND, but overall clusters 1 and 3, separately or combined, were significantly different from cluster 2. The t-test values computed using RMA values for all transcripts in cluster 1 vs. cluster 2, cluster 3 vs. cluster 2, and cluster 1+cluster 3 vs. cluster 2 were 1×10^−221^, 7.8×10^−25^, and 1.96×10^−24^, respectively. These results demonstrate that for the 2073 transcripts tested, gene expression in brain tissues of patients on ART tends to resemble that of uninfected subjects, indicating that ART is associated with a profound reduction in the extent of dysregulation of HAND-related genes in the brain. These results also suggest that the differences related to HIV-1 disease (and treatment) override potential differences resulting from genetic heterogeneity of individual donors.

The statistical similarity of ART transcriptomes with HIV-1-negative controls with respect to presumptive HIV-1-impacted genes (Figure 3A) prompted us to directly compare gene expression changes of ART brain tissue to expression in HAND brain tissue, without filtering microarray results through HIV-1-negative controls. This analysis inquires whether control of HIV-1 replication by ART removed viral perturbations to cellular gene expression in the brain, analogous to longitudinal studies of peripheral tissues from HIV-1-infected subjects pre and post-ART [24] which are not feasible for the brain. Using normalized RMA datasets for over 54,000 transcripts detected by the U133 chipset, we compared 7 treated patients to 8 untreated patients delimiting the results by FC of 1.5 and t-test value of 0.05; the complete list these differentially expressed transcripts is shown in Supplementary Table S6. In calculating the ratio of gene expression in ART to HAND, positive FC values in ART indicate an association of increased gene expression with treatment and negative FC indicate reduced cellular gene expression associated with treatment. Overall, we identified 640 significantly up-regulated and 276 down-regulated genes in this analysis (Supplementary Table S6). Samples from patients with treated HAND show increased expression (relative to untreated HAND) of many genes involved in neuronal functions including *synaptoporin* (SYNPR, FC 6.55 and p = 6.5×10^−4^), *neurofilament*, *light polypeptide* (NFEL, FC 6.15, p = 4.6×10^−4^), and *synaptotagmin IV* (SYT4, FC 4.16, p = 5.8×10^−4^) compared to samples from untreated patients. Conversely, genes involved in immune activation including *CD74 antigen* (CD74, FC −2.49, p-0.013), *complement component 1*, *q subcomponent*, *C chain* (C1QC, FC −2.47, p = 0.022), and *interferon-induced protein with tetratricopeptide repeats 2* (IFIT2, FC −2.35, p = 0.007) were reduced in expression in treated HAND patient samples relative to samples from untreated patients. For reference, Supplementary Table S6 also lists respective ART microarray data normalized to HIV-1-negative controls. Notably, all but 7 of the genes in treated patients that increased in expression relative to untreated HAND were unchanged in the ART/HIV-negative control comparison (Supplementary Table S6), suggesting that treated patients express these genes at normal (control) levels. Similar normalization of expression (272 out of 276) was found for genes that were down-modulated in ART versus HAND.

**Figure 4:**
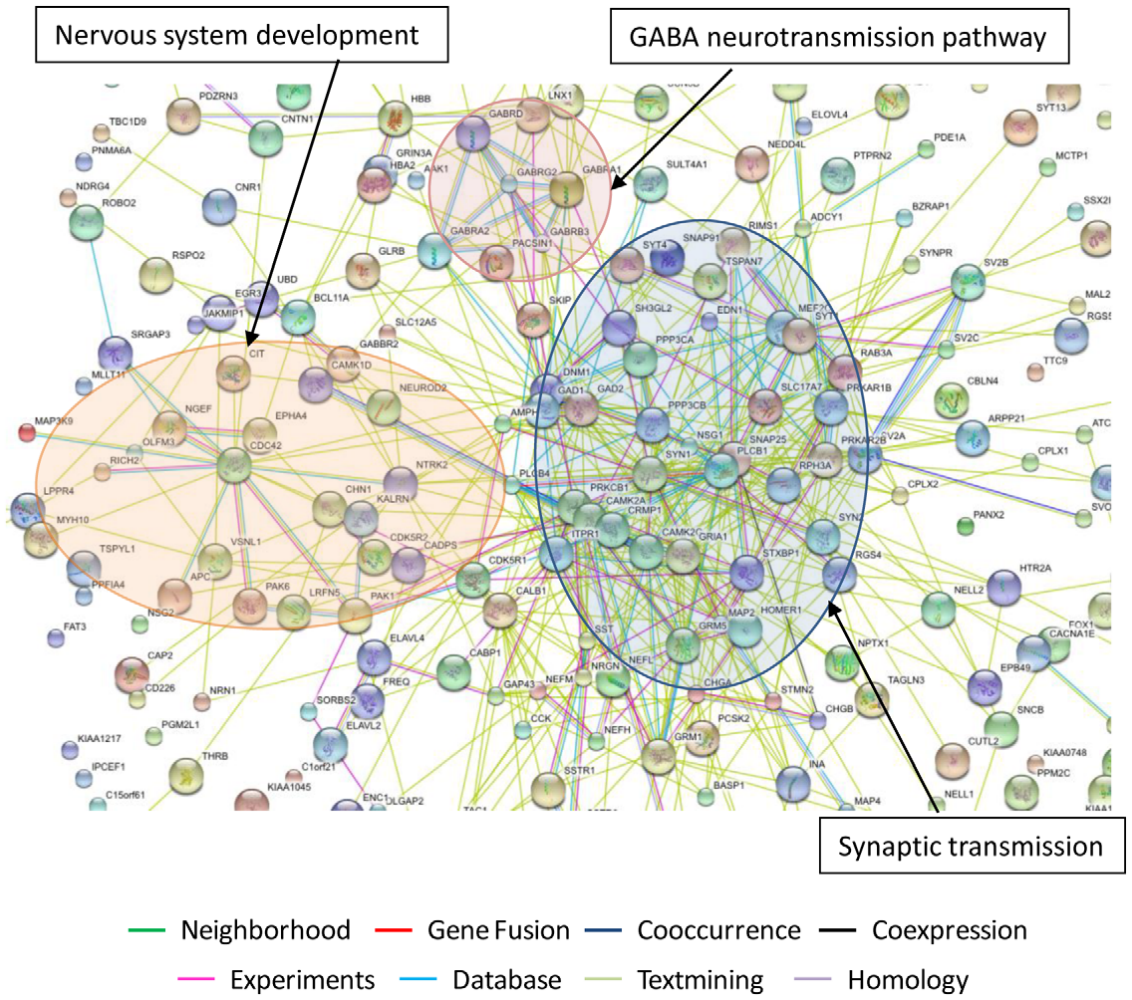
Predicted interaction networks of brain cell genes significantly downmodulated in untreated HAND and expressed at close to control levels in HAND patients under ART. The interactions between genes were identified using STRING software with each type of interaction distinguished by color. The most significantly regulated pathways identified in this analysis are synaptic transmission, nervous system development and GABA neurotransmission pathway.

To put these findings in the context of the biological pathways potentially affected by ART, we employed GAzer to compare the complete ART microarray datasets to those from HAND. Figure 3B depicts 16 biological processes that were most significantly changed in ART relative to HAND and Supplementary Table S7 provides complete statistics for this analysis. Twelve up-regulated processes in treated versus untreated HAND, with Z-scores ranging from 9.54 to 5.02, were related to neuronal function and repair. The down-modulated pathways included, in decreasing order of significance, immune responses, inflammatory responses, apoptosis, and responses to stress. These results suggest that ART reverses many dysfunctional processes of untreated HAND represented in gene ontology analysis. To provide an alternative view of the extensive up-regulation of cellular processes in ART relative to HAND, up-regulated genes with p-values of ≤0.01 included in Supplementary Table S6 were analyzed by STRING to identify predicted gene interaction networks (Figure 4). In this analysis ART was associated with improved synaptic transmission including synaptic vesicle system, nervous system development including cytoskeleton associated proteins, and GABA neurotransmission networks. These findings strongly suggest that by reducing HIV-1 replication, ART also reduces triggers to aberrant gene expression in the brain.

### Identification of genes that remain significantly dysregulated in the brain despite treatment

**Figure 5:**
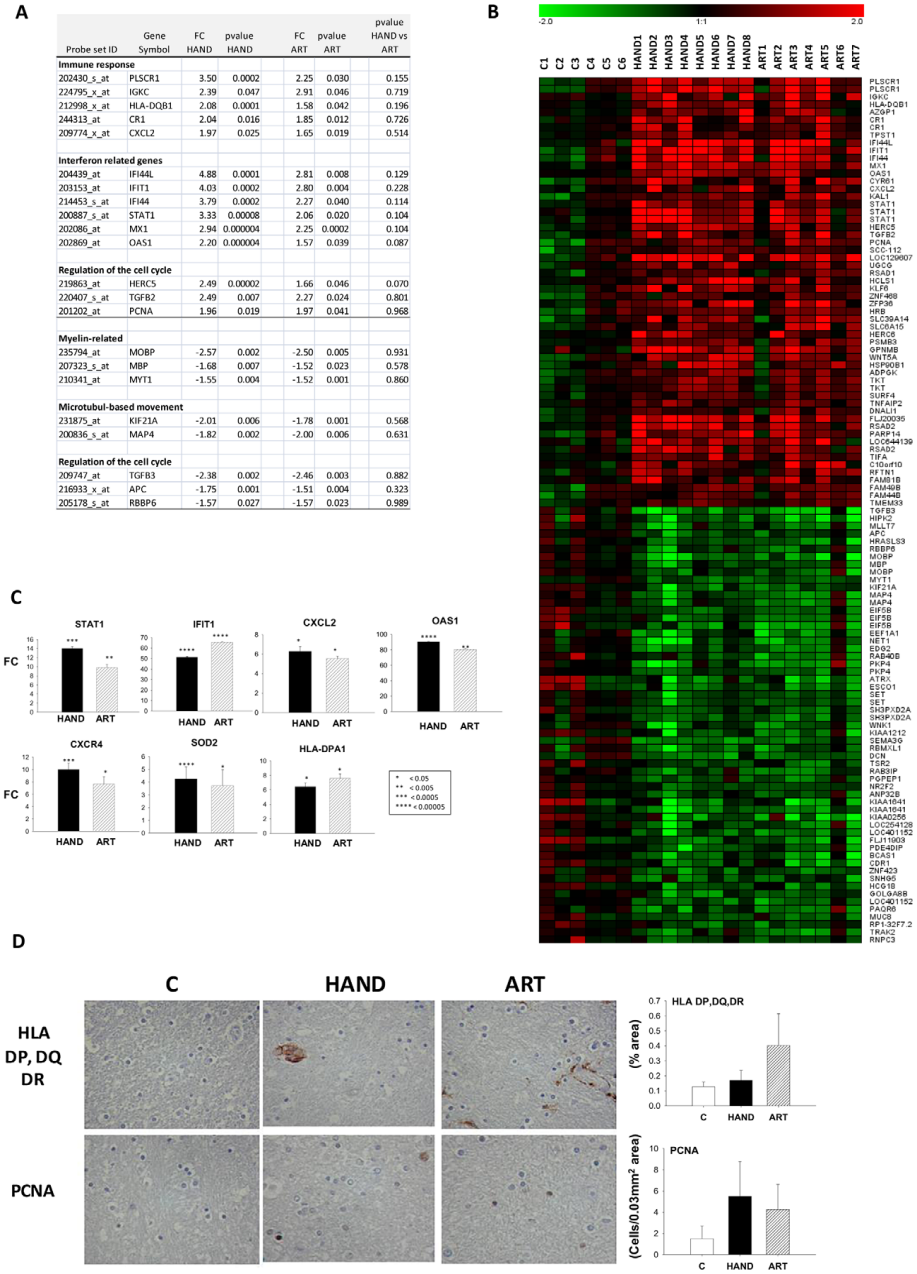
Common dysregulated genes in brain tissues of treated and untreated HAD patients. **A.** Selected genes significantly up or down-regulated in HAND or ART patients compared to uninfected subjects are tabulated (FC: Fold-change, pv: t-test p-value). **B.** Heatmap representation of significantly dysregulated genes in both HAND and ART datasets relative to uninfected subjects. **C.** QPCR analysis of expression of selected genes in HAND and ART patients relative to uninfected subjects. **D.** Immunostaining and quantitation of selected proteins in brain tissue.

The HAND patients in this study share cognitive dysfunction whether they were untreated or treated with ART (Table 1). To begin to identify transcripts that may contribute to HAND development or persistence despite ART, we used t-test to compare significantly changed genes in untreated (Supplementary Table S2) and treated (Supplementary Table S3) patients with HAND; a gene whose expression was not significantly different in the HAND versus ART comparison (p>0.05) was considered similarly dysregulated in both groups of patients relative to uninfected subjects. We have identified 43 such up-regulated and 42 down-regulated genes; they are listed grouped into biological categories in Supplemental Table S8, selected genes with their expression statistics are listed in Figure 5A, and their heatmap expression profiles in all subjects in this study are shown in Figure 5B. Considering functional characterization, genes related to immune responses were up-regulated in both HAND and ART samples including *complement receptor 1* (CR1), *chemokine*, *CXC motif*, *ligand 2* (CXCL2), *major histocompatibility complex*, *class II* HLA-DQB1 and interferon-mediated antiviral responses including *interferon-induced protein with tetratricopeptide repeats 1* (IFIT1); *interferon-induced protein 44* (IFI44); *myxovirus resistance 1* (MX1); *2′,5′-oligoadenylate synthetase 1* (OAS1), and *signal transducer and activator of transcription 1* (STAT1). Cell cycle pathway was dysregulated in both treated and untreated HAND patients, with some sets of genes up-regulated and others down-regulated (Figure 5A and 5B). Over-expression of selected transcripts in interferon or chemokine pathways in both HAND and ART brain samples was confirmed by real-time PCR (Figure 5C), over-expression of HLA Class II alleles and *proliferating cell nuclear antigen* (PCNA) was also demonstrated by immunohistochemistry (Figure 5D). Of particular interest, common down-regulated genes in treated and untreated patients with HAND included myelin-related genes *myelin-associated oligodendrocyte basic protein* (MOBP), *myelin transcription factor 1* (MYT1) and *myelin basic protein* (MBP). Down-regulation of MOBP and MYT1 was confirmed by real-time PCR, with the MYT1 gene being particularly suppressed in the ART group (FC = −38.38, p = 1×10^−5^) (Supplementary Table S5). This result is consistent with histopathological detection of myelin pallor in autopsy brain tissues from some patients with HAND [58], [59], although other explanations also exist [60].

### Correlation of gene expression changes in the brain and HIV-1 load in different compartments

**Figure 6:**
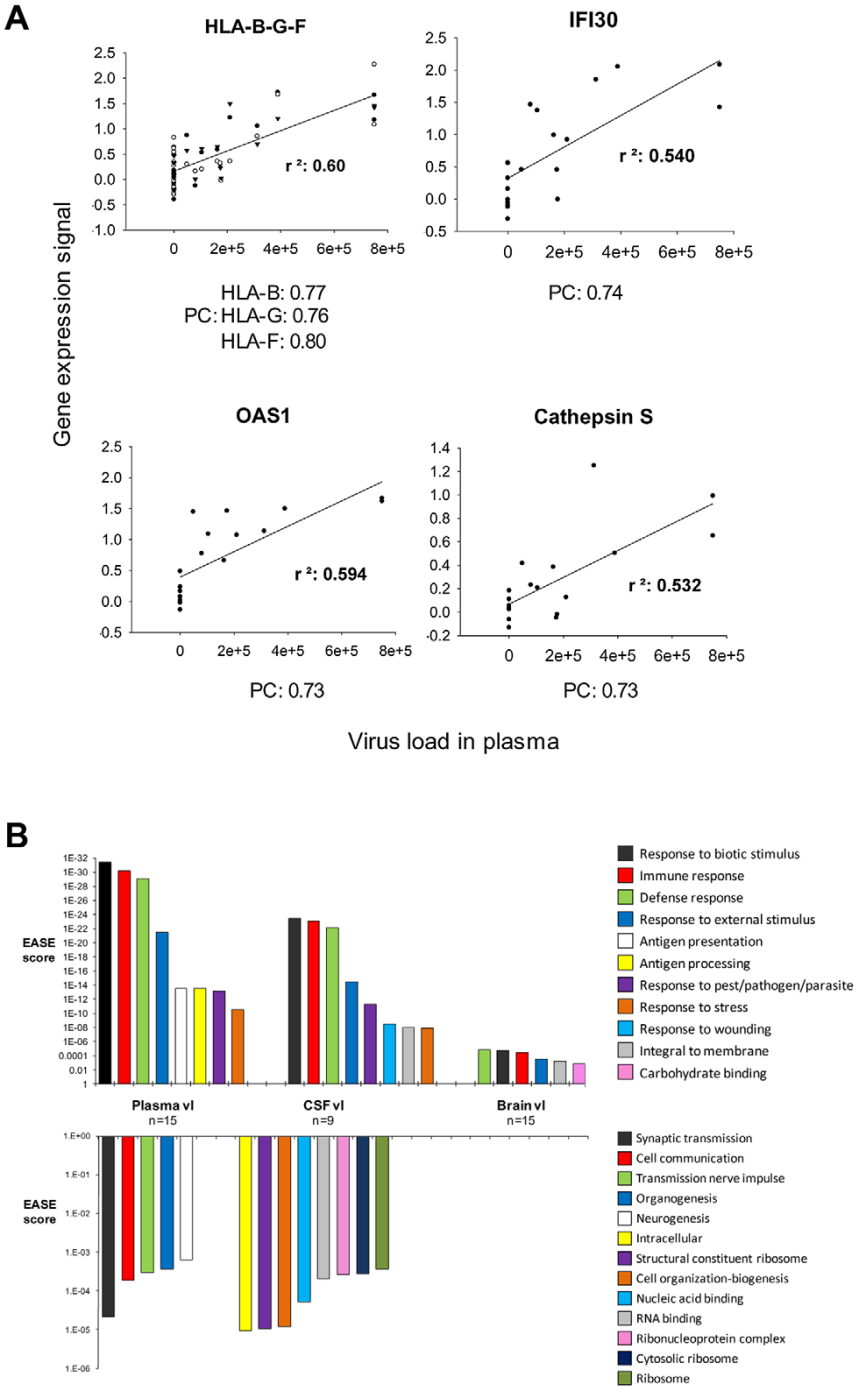
Global gene expression changes in HAND brain correlated the most with plasma and less with CSF and brain virus load. **A.** Examples of individual genes whose expression positively correlates with plasma virus load (r^2^: Coefficient of determination, PC: Pearson correlation). **B.** The upper panel shows positive correlations and the lower panel shows negative correlations with virus load (vl) in plasma, CSF, or brain respectively and gene expression in the brain. Genes that correlated positively and highly with virus load were analyzed using EASE software also negative correlations. The EASE score represent the significance of the regulated pathways.

Pearson's formula was applied to determine the correlation of the level of expression of each transcript with HIV-1 load in plasma, cerebrospinal fluid (CSF) and brain. Examples of genes correlating positively with plasma viral load are shown in Figure 6A, including histocompatibility loci HLA-B-G and F, *interferon-gamma-inducible protein 30* (IFI30), OAS1, and *Cathepsin S* (CTSS). Transcripts with a positive (>0.5) or negative (<−0.5) correlation with viral load were analyzed using the gene ontology software Expression Analysis Significance Explorer (EASE) to identify the pathways that correlated most with viral load (Figure 6B). Pathways positively correlated with viral load were similar in the three compartments (brain, CSF, plasma), including several immune activation responses. The main difference among the three compartments was the level of significance of the changes, as represented by the EASE score. All the pathways implicated in immune response correlated better with plasma viral load than with brain viral load. Even though CSF data were available for only 9 of the 15 infected patients, the positive correlations for CSF were higher than those for brain, although less strong than those observed for plasma. The categories of antigen presentation and processing were correlated only with viral load in plasma. Pathways down-regulated in correlation with viral load differed depending on the compartment tested. No biological pathway correlated negatively with brain viral load. Pathways negatively correlated with plasma viral load included synaptic transmission, cell communication, transmission of nerve impulse, organogenesis and neurogenesis. A wide variety of metabolic pathways negatively correlated with CSF viral load. Overall, grouping genes engaged in similar biological functions indicates that gene groups induced in the brain correlated best with virus burden in the periphery and that virus burden in the brain, unlike viral load in plasma or CSF, was uncorrelated to suppression of expression of any gene group.

## Discussion

We employed functional genomics to investigate the potential effects of antiretroviral treatment on brain pathophysiology in a cohort of patients who died with HAND. Our results suggest that ART profoundly, but not completely, alleviates aberrant gene expression in brain tissues of these patients. These findings may shed light on the molecular basis of HAND persistence despite treatment. Several points should be made about this work.

### Microarray profiles of HAND

The foundation of this work is a new comprehensive database of global gene expression profiles in brain tissues of patients with HAND. The profiles described here complement published datasets from previous array studies in HAND [41]–[44] with important differences. For the first time in this disease, we analyzed brain transcriptomes on the basis of the antiretroviral treatment of patients, revealing two distinct, largely non-overlapping groups of aggregate gene expression profiles termed ART and untreated HAND. The ART and untreated HAND profiles also formed separate clusters in unsupervised hierarchical cluster analysis [61] of individual patient datasets, confirming that they are phylogenetically distant from each other based on a large sets of aberrantly expressed genes used in this analysis (Figure 3A). Importantly, the hierarchical clustering distinction between treated and untreated patients was statistically more prominent than potential distinctions in gene expression related to other patient characteristics in our cohort including HIVE and intravenous drug use (Figure 3A and Supplementary Figure S2). Therefore, in most analyses in this work we considered datasets from patients with and without HIVE as one disease category, stratified only on the basis of ART. These results suggest that ART, through effects on HIV-1-associated changes in cellular gene expression [27], [62], [63], is one of the key biological variables governing the extent and pattern of gene dysregulation in molecular profiles of HIV-1 brain disease.

Another important difference with previous HAND array studies concerns the histological regions of the brain tested. We evaluated frontal deep white matter, whereas most of the previous studies focused on the neighboring cortical gray matter [41]–[44] or small gene sets in both brain regions [41]. White matter is the primary site of HIV-1 infection and HIV-1-associated neuropathologies [46], [47] and frontal cortex is one of the sites of synaptic and dendritic damage consequent to this infection [64], [65]. Transcriptomes from these two areas reflect different regional physiologies and therefore different aspects of HIV-1 neuropathogenesis. With these caveats, a meta-analysis summarized in Supplementary Table S9 identified a small number of genes involved in interferon-related responses (IFIT1, IFITM1, IFI44, MX1), synaptic functions (SYN1, SYN2, GABRG2, MAP2), and cell cycle (CDC42, CDK5R1, R2) that were dysregulated in common in the present and previously published HAND datasets. These genes and biological pathways may represent features of HIV-1-associated neuropathogenesis common to white and gray matter.

### Brain tissues of untreated patients with HIV-1 dementia show extensive dysregulation of gene expression independent of presence of HIVE

During the last twenty years, individual inflammatory and neurodegenerative mediators in HAND brains were demonstrated by immunocytochemistry, *in situ* hybridization, and other methods (reviewed in [66]–[69]). The bulk of this work was conducted with brain tissues from untreated patients, as brain autopsies after introduction of ART have become less frequent [70]. Consistent with these observations, the untreated HAND profile defined here reveals a broad and extensive dysregulation of cellular gene expression in brain tissues, with 1470 HAND-associated aberrantly expressed genes, up-regulation of immune activation, antiviral responses, and inflammation, and down-modulation of neuronal functions, neuronal repair, and cell cycle. It should be noted that our untreated HAND profile included transcriptomes from patients with and without HIVE (Figure 2 and 3). HIVE is a characteristic histopathology associated with high HIV-1 burdens in the brain [71], [72], and previous array studies documented differentially expressed transcripts and biological pathways potentially attributable to the extensive infection in the brain [43]–[45], [73]. We confirmed some of these differences in our untreated group with and without HIVE in gene ontology analysis but it is noteworthy that they differed mainly in the degree of dysregulation and not the biological pathways affected (Figure 1). Thus, at the level of a transcriptome analysis, the altered expression of some transcripts attributed to high HIV-1 burdens in HIVE [43], [44] contributes to but does not change the overall statistical characteristics of the molecular phenotype of HAND. Our results are consistent with reports of limited correlation between clinical manifestations of HAND and HIVE histopathology [58], [74], [75]. Rather, untreated HAND appears to correlate better with presence of inflammatory mediators and diffusely activated macrophages and microglial cells in the brain than with virus burdens in the tissue *per se* [17], [58], [75]–[78].

Independent microarray studies in other patient populations are needed to determine whether these gene expression profiles are fully representative of untreated HAND. In general, transcriptome profiles of disease can serve as a platform for verification of results obtained in studies of individual physiological processes [33], [79], [80] and as a tool for discovery of new ones. In this context, a number of “novel” (*i.e.*, relatively new to the HAND literature) dysregulated genes in our untreated HAND database may shed light on the process of HAND pathogenesis and merit further investigation. For example, transcripts encoding *apolipoprotein C-I* and *C-II* (APOC-I and APOC-II) were among the most up-regulated in the untreated HAND dataset, with FC of 5.71 (p = 9.7×10^−5^) and 3.35 (p = 0.009), respectively (Supplementary Table S2). Dysregulation of lipid metabolism linked to APOE polymorphism is a marker of AD [81] and was indicated in HIV-1 dementia [82], [83]. While we could not find reports on APOC-I in HAND, this lipoprotein was found in association with beta-amyloid plaques in AD brains and expression of human APOC-I allele in native APOC-I null mice was shown to impair learning and memory [84]. Conversely, *hemoglobin α-2* and *hemoglobin β* (HBA-2 and HBB) were among the most down-regulated transcripts in our untreated HAND dataset (FC of −5.31 and −4.22; Supplementary Tables S2 and S5). Neuronal hemoglobins are members of the globin superfamily which are predominantly expressed in neurons and may play an important role in neuroprotection [85]. Consistent with our findings, expression of neuronal hemoglobin is reduced or absent in disease affected brain regions in patients with several neurodegenerative conditions including AD and Parkinson's disease [86].

### ART efficiently mitigates aberrant gene expression in brain tissues of patients with HAND

The major finding of this work is the profound difference between the extensive global gene dysregulation observed in brain tissues of untreated patients with HAND and muted gene changes in their treated counterparts. The magnitude of ART effects in the brain suggested by our results was surprising given the limited clinical outcomes of ART on HAND [7], [87], including in the cohort evaluated here (Table 1), and variable findings in the CSF of treated patients [12]–[16]. The ART effects on the brain were inferred because we could not test brain RNA in the same individuals before and after initiation of therapy, as is possible in microarray studies with peripheral tissues [24], [27], [29], [31]. However, the overall effects of ART on altered gene expression were remarkably similar in the periphery and brain. This was evident in markedly fewer dysregulated genes in treated compared to untreated patients, in our case 253 versus 1470; and in a global shift in gene expression patterns from aberrant in the absence of treatment to muted dysregulation under treatment, for example in peripheral CD4^+^ T cells [27], [29], lymphoid tissue [26], and brain here (Figure 3A). Importantly, both in the CD4^+^ T lymphocyte study [27] and in the present work, gene expression profiles of treated patients were statistically similar to those of HIV-1-negative controls, suggesting a trend toward normalization of gene expression under ART. We confirmed this trend for selected gene products in the present work by real-time PCR and immunocytochemistry (Figure 2). These results suggest that ART regimens, which generally include at least one brain penetrant antiviral compound (Table 1), are similarly effective in mitigating global molecular changes in the brain and in peripheral tissues.

We noted two reciprocal effects of ART on gene dysregulation in brain tissues illuminating the systemic and brain-specific aspects of HAND pathogenesis. One is a significant and broad moderation of up-regulated genes linked to HIV-1 induced antiviral and inflammatory responses thought to drive HAND pathogenesis, including interferon-related ISG15 and IFIT3, macrophage markers CD68, CD163, and CD14, and chemokines and chemokine receptors CCL8, CCR1, and CXCR4 [67]–[69] (Figure 2 and 3). Interestingly, this effect of ART was common to diverse tissues examined by microarrays including brain (this study) and CD4^+^ T cells, macrophages, lymph nodes, and intestinal mucosa tested by others [24], [27], [29], [31], and thus it likely represents a system-wide response to suppression of HIV-1 replication. Although gene ontology pathways containing these genes in patients under ART were still up-regulated compared to controls (Figure 2B and Figure 5), our results suggest that ART can alleviate a surprisingly large number of deleterious responses in the brain that have been linked previously to HIV-1 infection in model systems [88]. This causal link is further strengthened by an apparent correlation in this work between treatment compliance and extent of gene dysregulation in the brain (Figure 2).

The other effect of ART in the brain we observed was specific to the nervous system and it involved normalization of a large number of down-regulated genes and biological pathways linked to nervous system functions and by extension to neurocognitive disease. For example, the bioinformatics tool STRING [89] identified nervous system development, synaptic transmission, and GABA-neurotransmission pathway as the three major predicted interaction networks of genes that approached normal expression in treated patients (Figure 4). On a smaller scale, our confirmatory tests showed that products such as MAP2 and *complexin-1* (CPLX1) were significantly down-regulated in untreated patients at RNA and protein levels and they were expressed at control-like levels in tissues from treated patients (Figure 2). It is conceivable that restoration of normal expression of at least some of these genes under ART would restore some aspects of normal brain physiology [7]. Although treated patients in our cohort still manifested HAND prior to death, our results suggest that they may have already shifted to a milder molecular profile in the brain that had more in common with HIV-1-negative controls than untreated patients.

Of interest, the global changes in brain cell gene expression seen by microarrays correlated positively with plasma but not brain virus burdens (Figure 6). This association may be analogous to the clustering of brain transcriptomes independently of encephalitis and brain HIV-1 burden by commonly dysregulated biological pathways. In any case, the single measurement of brain virus burden at autopsy may not capture the chronic insult to brain function suffered by patients living years with HIV-1 infection, albeit with some control exerted by ART.

### Genes dysregulated in common under ART and in untreated HAND

Perhaps the most intriguing contribution of the present array analysis lies in the ability to discern patterns of abnormality that persist in cognitively-impaired patients who are on central nervous system penetrant ART, and to distinguish these patterns from HAND in the untreated state. In the bioinformatics sense, we used ART as a biological filter to reduce the overall gene expression disturbance in brain transcriptomes of patients with HAND and through that determine whether continuing dysregulation of gene expression could play a role in continuing brain disease. The results indicate that continuing up-regulation of innate and adaptive immune responses are an important part of brain abnormalities in ART-treated dementia. Over-expression of Class II MHC in the brain persists despite therapy in our study; it has been associated with many neurodegenerative diseases [90]. Defects in myelin metabolism, shown for HAND at the gene expression level here and indicated previously by neuropathological observation of myelin pallor in brains of patients with HAD [58], are also common to many other neurodegenerative diseases [38], [91]. Activation of interferon-related genes often found in symptomatic HIV-1 infection may underlie abnormalities in cell function [28]. Such changes, coupled with cell cycle perturbations, may support emerging magnetic resonance spectroscopy studies that have demonstrated persistent white matter inflammation in patients with HIV-1-related cognitive impairment [92]. However, it is unclear what particular aspects of immune activation or response are relevant to nervous system dysfunction, and how to distinguish deleterious gene products from those that may function in a neuroprotective manner. In simian immunodeficiency virus infection, innate immunity, IL-6, and interferon responses are important elements in brain viral control [56], [93], but in a recent study expression of interferon-α in the brain was conclusively linked to neuronal dysfunction in a mouse model of HIVE [94]. Careful analysis of individual genes identified here may begin to clarify the mechanism of HAND persistence under treatment.

## Materials and Methods

### Ethics statement

Human brain samples and clinical data were obtained from the Manhattan HIV Brain Bank (MHBB), a member of the National NeuroAIDS Tissue Consortium, under an Institutional Review Board-approved protocol at the Mount Sinai School of Medicine. Written informed consent was obtained from all subjects in this study or their primary next-of-kin. HIV-1 and gene expression analyses were conducted on de-identified brain samples under an “exempt” status approved by an Institutional Review Board of St. Luke's-Roosevelt Hospital Center.

### Study subjects and brain regions used for analysis

Study subject information is listed in Table 1. Fifteen HIV-1-positive subjects used in this study were chosen on the basis of having HAND and the presence or absence of HIVE as determined by neuromedical or neuropsychological evaluation and postmortem neuropathology [95], and then were further categorized as either dying on or off ART. Fourteen of HIV-1-positive patients in this study died with HAD; one patient designated ART2 in Table 1 was classified as MND based on his lack of emotive/behavioral criteria [96]. Patients who displayed HAND without HIVE histopathology at autopsy met American Academy of Neurology criteria for HAND regardless of ART status. This definition requires demonstration of cognitive and functional impairments, and the presence of emotive or motoric phenomena [96]. Except for patient ART2, patients with HIVE were similarly impaired, regardless of ART status, or had histories of HAND on medical record review. Patients felt to have cognitive impairments) due to non-HIV-1 causes (neuropsychological impairment – other, as described in [95] were excluded. All patients dying on ART had substantive treatment histories, ranging 1 to 7 years prior to demise. The ART regimens at death had a mean duration of 16 months (range, 3 months to 3 years). For five of seven patients on ART, treatment compliance estimates were made by self-reported 4 day recall [97]. HIV-1-negative subjects were chosen on the basis of normal neurological function (as determined by chart review) and normal neurohistology. All neurohistologic diagnoses were rendered by a board-certified neuropathologist (SM) and a minimum of 50 sections were examined for each brain. The following brain pathology definitions were used in the present work (Table 1): Normal: no brain pathology; HIVE: HIV encephalitis; HIVE*: HIVE was limited to the basal ganglia (pid 30015); Minimal: minimal histopathological changes including trivial microscopic abnormalities such as an isolated vermal scar (pid 10119), a venous ectasia (pid 10063), atherosclerosis and minimal perivascular inflammation sub-threshold for diagnosis (pid 10001), and minimal perivascular inflammation sub-threshold for diagnosis (pid 10015). At the time of autopsy, coronal sections of brain were snap-frozen and maintained in −85°C until sub-dissection. Effort was made to keep the post mortem interval (PMI) to a minimum; the PMI for subjects in this study are listed in Table 1. Brain samples for this analysis were obtained from the centrum semiovale (deep white matter) at the coronal level of the genu of the corpus callosum. Multiple samples from the same region were dissected for gene expression profiling, real-time PCR (QPCR), and protein assays. Equivalent regions from the contralateral hemisphere were formalin fixed and utilized for immunohistochemistry.

### DNA and RNA isolation from human brain and reverse transcription

Total DNA and RNA were isolated from human brain tissue by, respectively, DNeasy Blood and Tissue Kit and RNeasy Mini Kit (Qiagen, Valencia, CA) according to the manufacturer's protocol. RNA was quantified by spectrophotometry and RNA quality was verified by spectrophotometry and agarose gel electrophoresis. RNA was then treated with DNAse I (Fisher Healthcare, Houston, TX). cDNA was synthesized using the Superscript First-Strand Kit (Invitrogen, Carlsbad, CA) for quantitative analysis and the WT-Ovation™ RNA Amplification System (NuGEN Technologies, Inc., San Carlos, CA) for relative analysis according to the manufacturer's protocol.

### Virus load in the brain

Viral RNA and DNA burdens in human brain were determined by quantitative real-time PCR using primers designed based on HIV-1 consensus sequences for the group of patients in this study. We first screened patient brain samples for the presence of HIV-1 content by a standard nested PCR for viral DNA or RNA (cDNA) as previously described [98]. To achieve broad detection of diverse Clade B HIV-1 species, first-round PCR was performed using custom designed primers for a conserved Clade B *gag* consensus sequences: SQ5 (+) 5′-CAA ATG GTA CAT CAG GCC ATA TCA CC-3′ and SQ3′ (−) 5′-CCC TGA CAT GCT GTC ATC ATT TCT TC-3′. For nested PCR step we used primers SQ5′ (above) and SK39 [99]; the PCR products were resolved on an agarose gel and detected by Southern Blot hybridization with (^32^P)-labeled probe SK19 [99] (Supplementary Figure S1). The HIV-1 *gag* nested PCR amplicons from individual HIV-1 DNA positive patients were sequenced and used to design patient consensus primers (5′ (+) QSQ5 5′-ACC CAT GTT T(T/A)C AGC ATT ATC AGA-3′ and 3′ (−) QSQ3 5′-GAT GTC CCC CCA CTG TGT TT-3′) and Taqman probe (HSQP 6FAM-AGC CAC CCC ACA AGA-MGBNFQ). For real-time PCR amplification, 2 µl of brain tissue DNA or 5 µl of cDNA were combined with 2× Universal Master Mix (Applied Biosystems, Carlsbad, CA), 900 nM consensus primer (custom synthesized by Invitrogen) and 200 nM probe (synthesized by Applied Biosystems; QPCR conditions were essentially as described [100]. Standard curve for viral DNA and cDNA quantification was constructed from graded amounts of HIV-1 NL4-3 plasmid DNA. Viral DNA burdens were normalized to total cellular DNA content by *β-globin* amplification and expressed as HIV-1 DNA copies/number of cells calculated from a *β-globin* DNA standard curve, with 2 copies of *β-globin* gene equaling one cell. Viral RNA burdens were normalized by tissue *glyceraldehydes-3-phosphate dehydrogenase* (GAPDH) content and expressed as number of viral copies in 1 µg tissue RNA [100].

### Microarray hybridization

Microarray experiments were conducted at the Bionomics Research and Technology Center in EOSHI University of Medicine and Dentistry of New Jersey. Total RNA were extracted from tissue samples using the RNeasy Mini Kit (Qiagen) followed by DNase I treatment. RNA qualities were assessed by electrophoresis using the Agilent Bioanalyzer 2100 and spectrophotometric analysis prior to cDNA synthesis. Fifty nanograms of total RNA from each sample were used to generate a high fidelity cDNA for array hybridization using NuGen WT-Ovation Pico RNA Amplification. Detailed protocols for sample preparation can be found at http://www.nugeninc.com. After fragmentation and biotin labeling using NuGen Encore Biotin Module, the samples were hybridized to Affymetrix Human Genome 133 plus 2.0 arrays. Washing and staining of all arrays were carried out in the Affymetrix fluidics module as per the manufacturer's protocol. The detection and quantitation of target hybridization was performed with an Affymetrix GeneChip Scanner. Data were assessed for array performance prior to analysis. The majority of patient samples were analyzed in duplicates starting from the cDNA synthesis step; in some cases second analysis was on an adjoining brain sample.

### Microarray data analysis

The .cel data files generated by the Affymetrix microarray hybridization platform were analyzed by the ArrayAssist software (Stratagene, Santa Clara, CA). Probe level analysis was performed using the RMA algorithm. After verification of data quality by Affymetrix internal controls and signal distribution analysis as described in Stratagene ArrayAssist Protocol, data was transformed using variance stabilization and logarithm transformation with a base of 2. Fluorescence values were normalized by mean intensities of all chip samples. Means of normalized expression values were calculated for duplicate samples from each individual, and these values were either used directly in some analytical programs (see below) or employed to calculate fold change (FC) in the transcript compared to HIV-1-negative controls. Genes showing FC values above 1.5 or below −1.5 and unpaired t-test p-values of <0.05 were defined as significantly changed. In some analyses we applied a t-test cutoff of p<0.01. To test for effect of antiretroviral treatment, we calculated the FC for significantly modulated transcripts separately from untreated and treated patients, the latter subdivided into all-treated and a subset without two known low-compliant patients, versus uninfected controls. We also compared directly normalized expression values of selected transcripts from treated and untreated patients to generate the treated versus untreated FC values that were not filtered through HIV-1-negative controls.

To compare brain samples of all the individuals included in the study we clustered the expression profiles using unsupervised Hierarchical Clustering (Average Linkage Clustering) and heatmap visualization software from Genesis [101] available at http://genome.tugraz.at/. This type of analysis allows us to identify the similarities and differences in the expression patterns of groups of patients and/or transcripts. For broad characterization of gene expression changes in untreated and treated patients we conducted gene set and gene ontology analysis using GAzer [50] (http://expressome.kobic.re.kr/GAzer/index.faces). GAzer is a web-based tool that identifies, by a parametric statistical analysis of complete primary normalized microarray data, over-represented sets of genes (functionally related genes) rather than individual genes. This type of analysis compensates for the fact that small changes not seen at the gene level are often detected when the gene set as a whole is examined [50], [51]. When analysis was limited to smaller sets of genes defined as differentially expressed, we performed functional categorization of gene families by EASE [102], available at the NIH web site (http://david.abcc.ncifcrf.gov/). The predicted biological pathway and network relationships among differentially expressed genes in our array datasets were identified using the Search Tool for the Retrieval if Interacting Genes/Proteins (STRING) (http://string-db.org/) [89].

### Gene expression validation by QPCR

Changes in expression of selected genes identified by microarrays analysis were validated in the same or adjoining brain samples by QPCR using Taqman chemistry and probes from the Universal Probe Library (Roche, Indianapolis, IN). Primers were designed using the online ProbeFinder software available at the Roche Universal Probe Library Assay Design Center (http://www.roche-applied-science.com). The QPCR reactions contained 2 µl of cDNA generated from tissue RNA obtained as described above, 10 µl of 2× Universal Master Mix (Applied Biosystems-ABI), 0.2 µl of each forward and reverse primers at 200 nM, 0.2 µl of probe at 100 nM, and RNAse/DNAase-free water. All reactions were performed in duplicate and were run in a 7500 real-time PCR system (ABI). Raw data was analyzed using the 7500 System SDS Software (ABI). Data was normalized using 2 housekeeping genes, GAPDH and *ribosomal protein S18* (RPS18) to assure reproducibility. Relative quantification employed the comparative threshold cycle method (Applied Biosystems Technical Bulletin n°2).

### Immunohistochemistry

For immunohistochemical analysis, formalin fixed blocks were taken from the frontal white matter of the autopsy brains of 4 normal, 6 HIV-1-non-treated, and 4 HIV-1-ART-treated individuals. A microarray block consisting of 3 tissue punches diameter of 1 mm) from each block was constructed. 5 µM serial sections were cut and immunohistochemistry performed with an array of antibodies listed in the Table 2.

**Table 2:** Antibodies used for immunohistochemistry.

Antigen	Dilution	Antibody Type	Source
C3c complement	1∶3000	Rabbit polyclonal	DakoCytomation
CD45, leukocyte common antigen	1∶100	Mouse monoclonal	DakoCytomation
CD68/KP1	1∶1000	Mouse monoclonal	DakoCytomation
HLA-DP, DQ, DR	1∶500	Mouse monoclonal	DakoCytomation
MAP2	1∶200	Mouse monoclonal	Sigma
PCNA	1∶1000	Mouse monoclonal	DakoCytomation

Formalin-fixed, paraffin-embedded sections were deparaffinized with xylenes, hydrated in graded alcohols, and incubated with 3% H_2_O_2_ in methanol. Following washing, sections were boiled in Target retrieval solution (DAKO Corp., Carpinteria, CA), subsequently incubated in a serum free protein blocking solution and then incubated with the primary antibodies indicated in Table 2. CD45 (PTPRC) was incubated overnight at 4°C and the all the remaining antibodies for 1 h at room temperature. Primary antibodies were detected with peroxidase anti rabbit or mouse IgG ImmPRESS (DAKO Corp.) reagent and counterstained with hematoxylin. Slides were visualized in a light microscope and either one, three or six 0.03 mm^2^ areas of white matter staining from each case were photographed using a 40× objective and a Nikon Coolpix II digital camera attached to the microscope by a Coolpix MDC lens. The intensity of illumination and position of sub-stage condenser on the microscope were constant for all images. The number of areas taken depended on the type of quantitative analysis that followed. The percentage area occupied by cells immunoreactive for CD68, CD45 and HLA DP, DQ, DR was quantitated by analysis of six 0.03 mm^2^ images using a proprietary automated morphometric analysis software [103]. The mean intensity of MAP2 and STAT1 staining was analyzed on one 0.03 mm^2^ image, utilizing Image J software (http://rsbweb.nih.gov/ij/). Each image was converted to 8-bit grayscale and a mean gray value of the pixels in the full photographic image was measured after a background subtraction and inversion were performed. For C3c and PCNA, cell counts were done by eye using either three 0.03 mm^2^ (C3c) or the full punch (PCNA). Statistical analysis was performed using StatView (V.5.0.1) (Adept Scientific, Bethesda, MD). The principal statistical test used was the Analysis of Variance for single comparisons (ANOVA). Follow-up post-hoc tests were conducted when required. Significance values were set at 0.05 and below.

### Correlation analysis

Correlation analysis between virus loads in plasma, CSF, or brain and gene expression in the brain was performed using the Pearson's correlation formula in Microsoft Excel software (Microsoft Corporation, Redmond, WA). Transcripts showing positive (>0.5) or negative correlation (<−0.5) were categorized into biological functional pathways using EASE software.

### Microarray data repository

The microarray results presented here are available in the Gene Expression Omnibus database (www.ncbi.nlm.nih.gov/geo) in a MIAME compliant format under accession number GSE28160.

### Gene accession numbers

The Gene ID numbers for the genes mentioned in the text are listed below. The ID number corresponds to the ‘National Center for Biotechnology Information database’ (http://www.ncbi.nlm.nih.gov/gene): CD4 (920), C3 (718), CD68 (968), PTPRC (5788), CDK5R2 (8941), MAP2 (4133), CPLX1 (10815), SYNPR (132204), NFEL (4747), SYT4 (6860), CD74 (972), C1QC (714), IFIT2 (3433), CR1 (1378), CXCL2 (2920), HLA-DQB1 (3119), IFIT1 (3434), IFI44 (10561), MX1 (4599), OAS1 (4938), STAT1 (6772), PCNA (5111), MOBP (4336), MYT1 (4661), MBD (4155), IFI30 (10437), CTSS (1520), IFITM1 (8519), B2M (567), CD14 (929), APOC-I (341), APOC-II (344), HBA-2 (3040), HBB (3043), IFI16 (3428), TLR7 (51284), SYN1 (6853), SYN2 (6854), GABRG2 (2566), CDC42 (998), CDK5R1 (8851), GAPDH (2597), RPS18 (6222).

## Supporting Information

Figure S1 **HIV-1 brain burdens of study subjects.** **A.** Analysis of HIV-1 brain burdens in patient tissues by real-time PCR. Control: uninfected patients; HAND: patients with HAND without HIVE; HAND/HIVE: patients with HAND and HIVE; ART: treated patients of either category. HIV-1 DNA copies were prorated per 500,000 cells and HIV-1 RNA copies were per 1 µg RNA. HIV-1 plasma burdens are shown for comparison. **B.** Analysis of HIV-1 brain burdens by traditional PCR amplification and Southern blot hybridization. *β-globin* was used for normalization. For details, see Materials and Methods.
(TIF)

Figure S2 **Hierarchical cluster analysis of study subjects.** The Figure represents enlarged image of the cluster panel shown on the top of Figure 3A. The cluster tree shows computer-generated phenotypic relationship between the subjects in the study according to the presence and relative expression of 2073 HAND-associated transcripts listed in Supplementary Table S2. Note close phenotypic relationship between 7 out of 8 untreated HAND distinct from treated patients and controls. Treated patients clustered mostly together in two inter-related clusters with HIV-1-negative controls,
(TIF)

Table S1 **Comparison of HAND and HAND/HIVE GO pathways.** The Table shows an extended list of significantly altered GO pathways in untreated HAND and HAND/HIVE datasets as defined by GAzer software. Significance of change in GO pathways was determined by Z-score, p-and q-values, and Bonferroni correction value; the data shown is delimited by Z-scores.
(XLS)

Table S2 **Gene changes in untreated HAND.** The Table shows a complete list of significantly changed transcripts and genes in brain tissues from untreated HAND patients compared to HIV-1 negative controls. FC: Fold-change, pv: t-test p-value.
(XLS)

Table S3 **Gene changes in treated HAND.** The Table shows a complete list of significantly changed transcripts and genes in brain tissues from treated HAND patients compared to HIV-1 negative controls. FC: Fold-change, pv: t-test p-value.
(XLS)

Table S4 **HAND and ART GO pathways vs. controls.** The Table shows an extended list of significantly altered GO pathways in untreated HAND vs. control (sheet 1), ART vs. control (sheet 2), and ARTa vs. control (sheet 3); all as defined by Gazer software. Significance of change in GO pathways was determined by Z-score, p-and q-values, and Bonferroni correction value; the data shown is delimited by Z-scores.
(XLS)

Table S5 **List of genes validated by real-time PCR.** The Table shows average of 2 independent QPCR experiments for each gene listed; microarray results are shown for comparison. QPCR was conducted in duplicates on tissue samples adjoining to those used for microarray analysis; Taqman chemistry was employed as described in Materials and Methods.
(PPT)

Table S6 **Direct comparison of ART and HAND gene expression datasets.** The Table shows significantly changed transcripts and genes derived by direct comparison of RMA values from complete ART and HAND microarray datasets, without prior normalization to HIV-1 negative controls. Also shown are expression values for the same genes in ART vs. HIV-1 negative control. Separate tabs for up- and down-regulated genes.
(XLS)

Table S7 **Comparison of ART and HAND gene expression datasets for GO pathways.** Data was analyzed as in Table S6 but using GAzer software to define gene ontology pathways. Significance of change in GO pathways was determined by Z-score, p-and q-values, and Bonferroni correction value; the data shown is delimited by Z-scores.
(XLS)

Table S8 **Common significantly changed genes in treated and untreated patients.** A gene whose expression was not significantly different in the HAND versus ART comparison (p>0.05) was considered similarly dysregulated in both groups of patients relative to uninfected subjects. FC: Fold-change, pv: t-test p-value.
(XLS)

Table S9 **Gene expression commonalities with other array studies of brain tissues from patients with HAND.**
(PPTX)

## References

[1] Antinori A, Arendt G, Becker JT, Brew BJ, Byrd DA (2007). Updated research nosology for HIV-associated neurocognitive disorders.. Neurology.

[2] Chang L, Ernst T, Leonido-Yee M, Witt M, Speck O (1999). Highly active antiretroviral therapy reverses brain metabolite abnormalities in mild HIV dementia.. Neurology.

[3] Robertson KR, Robertson WT, Ford S, Watson D, Fiscus S (2004). Highly active antiretroviral therapy improves neurocognitive functioning.. J Acquir Immune Defic Syndr.

[4] Robertson KR, Smurzynski M, Parsons TD, Wu K, Bosch RJ (2007). The prevalence and incidence of neurocognitive impairment in the HAART era.. AIDS.

[5] Sacktor N, McDermott MP, Marder K, Schifitto G, Selnes OA (2002). HIV-associated cognitive impairment before and after the advent of combination therapy.. J Neurovirol.

[6] Tozzi V, Balestra P, Bellagamba R, Corpolongo A, Salvatori MF (2007). Persistence of neuropsychologic deficits despite long-term highly active antiretroviral therapy in patients with HIV-related neurocognitive impairment: prevalence and risk factors.. J Acquir Immune Defic Syndr.

[7] Ellis R, Langford D, Masliah E (2007). HIV and antiretroviral therapy in the brain: neuronal injury and repair.. Nat Rev Neurosci.

[8] Harezlak J, Buchthal S, Taylor M, Schifitto G, Zhong J (2011). Persistence of HIV-associated cognitive impairment, inflammation, and neuronal injury in era of highly active antiretroviral treatment.. AIDS.

[9] Simioni S, Cavassini M, Annoni JM, Rimbault Abraham A, Bourquin I (2010). Cognitive dysfunction in HIV patients despite long-standing suppression of viremia.. AIDS.

[10] Heaton RK, Clifford DB, Franklin DR, Woods SP, Ake C (2010). HIV-associated neurocognitive disorders persist in the era of potent antiretroviral therapy: CHARTER Study.. Neurology.

[11] McArthur JC (2004). HIV dementia: an evolving disease.. J Neuroimmunol.

[12] Chang L, Ernst T, St Hillaire C, Conant K (2004). Antiretroviral treatment alters relationship between MCP-1 and neurometabolites in HIV patients.. Antivir Ther.

[13] Cysique L, Brew BJ, Halman M, Catalan J, Sacktor N (2005). Undetectable cerebrospinal fluid HIV RNA and beta-2 microglobulin do not indicate inactive AIDS dementia complex in highly active antiretroviral therapy-treated patients.. J Acquir Immune Defic Syndr.

[14] Edén A, Price RW, Spudich S, Fuchs D, Hagberg L (2007). Immune activation of the central nervous system is still present after >4 years of effective highly active antiretroviral therapy.. J Infect Dis.

[15] McArthur JC, McDermott MP, McClernon D, St Hillaire C, Conant K (2004). Attenuated central nervous system infection in advanced HIV/AIDS with combination antiretroviral therapy.. Arch Neurol.

[16] Neuenburg JK, Furlan S, Bacchetti P, Price RW, Grant RM (2005). Enrichment of activated monocytes in cerebrospinal fluid during antiretroviral therapy.. AIDS.

[17] Anthony IC, Ramage SN, Carnie FW, Simmonds P, Bell JE (2005). Influence of HAART on HIV-related CNS disease and neuroinflammation.. J Neuropathol Exp Neurol.

[18] Bild AH, Yao G, Chang JT, Wang Q, Potti A (2006). Oncogenic pathway signatures in human cancers as a guide to targeted therapies.. Nature.

[19] Golub TR, Slonim DK, Tamayo P, Huard C, Gaasenbeek M (1999). Molecular classification of cancer: class discovery and class prediction by gene expression monitoring.. Science.

[20] Köbel M, Kalloger SE, Boyd N, McKinney S, Mehl E (2008). Ovarian carcinoma subtypes are different diseases: implications for biomarker studies.. PLoS Med.

[21] Parmigiani G, Garrett-Mayer ES, Anbazhagan R, Gabrielson E (2004). A cross-study comparison of gene expression studies for the molecular classification of lung cancer.. Clin Cancer Res.

[22] Rhodes DR, Yu J, Shanker K, Deshpande N, Varambally R (2004). Large-scale meta-analysis of cancer microarray data identifies common transcriptional profiles of neoplastic transformation and progression.. Proc Natl Acad Sci USA.

[23] Stratford JK, Bentrem DJ, Anderson JM, Fan C, Volmar KA (2010). A six-gene signature predicts survival of patients with localized pancreatic ductal adenocarcinoma.. PLoS Med.

[24] Li Q, Schacker T, Carlis J, Beilman G, Nguyen P (2004). Functional genomic analysis of the response of HIV-1-infected lymphatic tissue to antiretroviral therapy.. J Infect Dis.

[25] Li Q, Smith AJ, Schacker TW, Carlis JV, Duan L (2009). Microarray analysis of lymphatic tissue reveals stage-specific, gene expression signatures in HIV-1 infection.. J Immunol.

[26] Smith AJ, Li Q, Wietgrefe SW, Schacker TW, Reilly CS (2010). Host genes associated with HIV-1 replication in lymphatic tissue.. J Immunol.

[27] Rotger M, Dang KK, Fellay J, Heinzen EL, Feng S (2010). Genome-wide mRNA expression correlates of viral control in CD4+ T-cells from HIV-1-infected individuals.. PLoS Pathog.

[28] Wu JQ, Dwyer DE, Dyer WB, Yang YH, Wang B (2008). Transcriptional profiles in CD8+ T cells from HIV+ progressors on HAART are characterized by coordinated up-regulation of oxidative phosphorylation enzymes and interferon responses.. Virology.

[29] Van den Bergh R, Florence E, Vlieghe E, Boonefaes T, Grooten J (2010). Transcriptome analysis of monocyte-HIV interactions.. Retrovirology.

[30] Richard Y, Amiel C, Jeantils V, Mestivier D, Portier A (2010). Changes in blood B cell phenotypes and Epstein-Barr virus load in chronically human immunodeficiency virus-infected patients before and after antiretroviral therapy.. J Infect Dis.

[31] Guadalupe M, Sankaran S, George MD, Reay E, Verhoeven D (2006). Viral suppression and immune restoration in the gastrointestinal mucosa of human immunodeficiency virus type 1-infected patients initiating therapy during primary or chronic infection.. J Virol.

[32] Sun B, Abadjian L, Rempel H, Calosing C, Rothlind J (2010). Peripheral biomarkers do not correlate with cognitive impairment in highly active antiretroviral therapy-treated subjects with human immunodeficiency virus type 1 infection.. J Neurovirol.

[33] Glanzer JG, Haydon PG, Eberwine JH (2004). Expression profile analysis of neurodegenerative disease: advances in specificity and resolution.. Neurochem Res.

[34] Blalock EM, Geddes JW, Chen KC, Porter NM, Markesbery WR (2004). Incipient Alzheimer's disease: microarray correlation analyses reveal major transcriptional and tumor suppressor responses.. Proc Natl Acad Sci USA.

[35] Lukiw WJ (2004). Gene expression profiling in fetal, aged, and Alzheimer hippocampus: a continuum of stress-related signaling.. Neurochem Res.

[36] Miller RM, Federoff HJ (2006). Microarrays in Parkinson's disease: a systematic approach.. NeuroRx.

[37] Sutherland GT, Matigian NA, Chalk AM, Anderson MJ, Silburn PA (2009). A cross-study transcriptional analysis of Parkinson's disease.. PLoS One.

[38] Hakak Y, Walker JR, Li C, Wong WH, Davis KL (2001). Genome-wide expression analysis reveals dysregulation of myelination-related genes in chronic schizophrenia.. Proc Natl Acad Sci USA.

[39] Lock C, Hermans G, Pedotti R, Brendolan A, Schadt E (2002). Gene-microarray analysis of multiple sclerosis lesions yields new targets validated in autoimmune encephalomyelitis.. Nat Med.

[40] Gebicke-Haerter PJ (2005). Microarrays and expression profiling in microglia research and in inflammatory brain disorders.. J Neurosci Res.

[41] Gelman BB, Soukup VM, Schuenke KW, Keherly MJ, Holzer C (2004). Acquired neuronal channelopathies in HIV-associated dementia.. J Neuroimmunol.

[42] Shapshak P, Duncan R, Torres-Muñoz JE, Duran EM, Minagar A (2004). Analytic approaches to differential gene expression in AIDS versus control brains.. Front Biosci.

[43] Masliah E, Roberts ES, Langford D, Everall I, Crews L (2004). Patterns of gene dysregulation in the frontal cortex of patients with HIV encephalitis.. J Neuroimmunol.

[44] Everall I, Salaria S, Roberts E, Corbeil J, Sasik R (2005). Methamphetamine stimulates interferon inducible genes in HIV infected brain.. J Neuroimmunol.

[45] Tatro ET, Scott ER, Nguyen TB, Salaria S, Banerjee S (2010). Evidence for Alteration of Gene Regulatory Networks through MicroRNAs of the HIV-infected brain: novel analysis of retrospective cases.. PLoS One.

[46] Lopez-Villegas D, Lenkinski RE, Frank I (1997). Biochemical changes in the frontal lobe of HIV-infected individuals detected by magnetic resonance spectroscopy.. Proc Natl Acad Sci USA.

[47] Sailasuta N, Shriner K, Ross B (2009). Evidence of reduced glutamate in the frontal lobe of HIV-seropositive patients.. NMR Biomed.

[48] Langford D, Marquie-Beck J, de Almeida S, Lazzaretto D, Letendre S (2006). Relationship of antiretroviral treatment to postmortem brain tissue viral load in human immunodeficiency virus-infected patients.. J Neurovirol.

[49] Wang Z, Trillo-Pazos G, Kim SY, Canki M, Morgello S (2004). Effects of human immunodeficiency virus type 1 on astrocyte gene expression and function: potential role in neuropathogenesis.. J Neurovirol.

[50] Kim S-B, Yang S, Kim S-K, Kim SC, Woo HG (2007). GAzer: gene set analyzer.. Bioinformatics.

[51] Kim SY, Volsky DJ (2005). PAGE: parametric analysis of gene set enrichment.. BMC Bioinformatics.

[52] Fischer-Smith T, Croul S, Sverstiuk AE, Capini C, L'Heureux D (2001). CNS invasion by CD14+/CD16+ peripheral blood-derived monocytes in HIV dementia: perivascular accumulation and reservoir of HIV infection.. J Neurovirol.

[53] McManus CM, Weidenheim K, Woodman SE, Nunez J, Hesselgesser J (2000). Chemokine and chemokine-receptor expression in human glial elements: induction by the HIV protein, Tat, and chemokine autoregulation.. Am J Pathol.

[54] Moore DJ, Masliah E, Rippeth JD, Gonzalez R, Carey CL (2006). Cortical and subcortical neurodegeneration is associated with HIV neurocognitive impairment.. AIDS.

[55] Yang B, Singh S, Bressani R, Kanmogne GD (2010). Cross-talk between STAT1 and PI3K/AKT signaling in HIV-1-induced blood-brain barrier dysfunction: Role of CCR5 and implications for viral neuropathogenesis.. J Neurosci Res.

[56] Roberts ES, Burudi EME, Flynn C, Madden LJ, Roinick KL (2004). Acute SIV infection of the brain leads to upregulation of IL6 and interferon-regulated genes: expression patterns throughout disease progression and impact on neuroAIDS.. J Neuroimmunol.

[57] Logan JMJ, Edwards KJ, Logan J, Edwards K, Saunders N (2009). An overview of PCR platforms.. Real-Time PCR: Current Technology and Applications.

[58] Glass JD, Wesselingh SL, Selnes OA, McArthur JC (1993). Clinical-neuropathologic correlation in HIV-associated dementia.. Neurology.

[59] Wohlschlaeger J, Wenger E, Mehraein P, Weis S (2009). White matter changes in HIV-1 infected brains: a combined gross anatomical and ultrastructural morphometric investigation of the corpus callosum.. Clin Neurol Neurosurg.

[60] Power C, Kong PA, Crawford TO, Wesselingh S, Glass JD (1993). Cerebral white matter changes in acquired immunodeficiency syndrome dementia: alterations of the blood-brain barrier.. Ann Neurol.

[61] Kapetanovic IM, Rosenfeld S, Izmirlian G (2004). Overview of commonly used bioinformatics methods and their applications.. Ann NY Acad Sci.

[62] Chun TW, Justement JS, Lempicki RA, Yang J, Dennis G (2003). Gene expression and viral prodution in latently infected, resting CD4+ T cells in viremic versus aviremic HIV-infected individuals.. Proc Natl Acad Sci USA.

[63] van't Wout AB, Lehrman GK, Mikheeva SA, O'Keeffe GC, Katze MG (2003). Cellular gene expression upon human immunodeficiency virus type 1 infection of CD4^+^-T-cell lines.. J Virol.

[64] Budka H (1991). Neuropathology of human immunodeficiency virus infection.. Brain Pathol.

[65] Everall I, Luthert P, Lantos P (1993). A review of neuronal damage in human immunodeficiency virus infection: its assessment, possible mechanism and relationship to dementia.. J Neuropathol Exp Neurol.

[66] Kolson DL, Pomerantz RJ (1996). AIDS dementia and HIV-1-induced neurotoxicity: possible pathogenic associations and mechanisms.. J Biomed Sci.

[67] Kaul M, Garden GA, Lipton SA (2001). Pathways to neuronal injury and apoptosis in HIV-associated dementia.. Nature.

[68] Nath A (2002). Human immunodeficiency virus (HIV) proteins in neuropathogenesis of HIV dementia.. J Infect Dis.

[69] Kraft-Terry SD, Buch SJ, Fox HS, Gendelman HE (2009). A coat of many colors: neuroimmune crosstalk in human immunodeficiency virus infection.. Neuron.

[70] Scaravilli F, Bazille C, Gray F (2007). Neuropathologic contributions to understanding AIDS and the central nervous system.. Brain Pathol.

[71] Sharer LR, Saito Y, Epstein LG, Blumberg BM (1994). Detection of HIV-1 DNA in pediatric AIDS brain tissue by two-step ISPCR.. Adv Neuroimmunol.

[72] Navia BA, Cho ES, Petito CK, Price RW (1986). The AIDS dementia complex: II. Neuropathology.. Ann Neurol.

[73] Roberts ES, Zandonatti MA, Watry DD, Madden LJ, Henriksen SJ (2003). Induction of pathogenic sets of genes in macrophages and neurons in NeuroAIDS.. Am J Pathol.

[74] Bell JE (1998). The neuropathology of adult HIV infection.. Rev Neurol (Paris).

[75] Gray F, Adle-Biassette H, Chretien F, Lorin de la Grandmaison G, Force G (2001). Neuropathology and neurodegeneration in human immunodeficiency virus infection. Pathogenesis of HIV-induced lesions of the brain, correlations with HIV-associated disorders and modifications according to treatments.. Clin Neuropathol.

[76] Kraft-Terry SD, Stothert AR, Buch S, Gendelman HE (2010). HIV-1 neuroimmunity in the era of antiretroviral therapy.. Neurobiol Dis.

[77] Xing HQ, Hayakawa H, Gelpi E, Kubota R, Budka H (2009). Reduced expression of excitatory amino acid transporter 2 and diffuse microglial activation in the cerebral cortex in AIDS cases with or without HIV encephalitis.. J Neuropathol Exp Neurol.

[78] Yadav A, Collman RG (2009). CNS inflammation and macrophage/microglial biology associated with HIV-1 infection.. J Neuroimmune Pharmacol.

[79] D'Agata V, Cavallaro S (2004). Genomic portraits of the nervous system in health and disease.. Neurochem Res.

[80] Hoheisel JD (2006). Microarray technology: beyond transcript profiling and genotype analysis.. Nat Rev Genet.

[81] Stefani M, Liguri G (2009). Cholesterol in Alzheimer's disease: unresolved questions.. Curr Alzheimer Res.

[82] Cutler RG, Haughey NJ, Tammara A, McArthur JC, Nath A (2004). Dysregulation of sphingolipid and sterol metabolism by ApoE4 in HIV dementia.. Neurology.

[83] Haughey NJ, Bandaru VV, Bae M, Mattson MP (2010). Roles for dysfunctional sphingolipid metabolism in Alzheimer's disease neuropathogenesis.. Biochim Biophys Acta.

[84] Abildayeva K, Berbee JF, Blokland A, Jansen PJ, Hoek FJ (2008). Human apolipoprotein C-I expression in mice impairs learning and memory functions.. J Lipid Res.

[85] Hua S, Antao ST, Corbett A, Witting PK (2010). The significance of neuroglobin in the brain.. Curr Med Chem.

[86] Ferrer I, Gomez A, Carmona M, Huesa G, Porta S (2011). Neuronal hemoglobin is reduced in Alzheimer's disease, argyrophilic grain disease, Parkinson's disease, and dementia with Lewy bodies.. J Alzheimers Dis.

[87] Valcour V, Sithinamsuwan P, Letendre S, Ances B (2011). Pathogenesis of HIV in the central nervous system.. Curr HIV/AIDS Rep.

[88] Lipton S, Gendelman HE (1995). Dementia associated with the acquired immunodeficiency syndrome.. N Engl J Med.

[89] Jensen LJ, Kuhn M, Stark M, Chaffron S, Creevey C (2009). STRING 8–a global view on proteins and their functional interactions in 630 organisms.. Nucl Acids Res.

[90] Piehl F, Olsson T (2009). Inflammation and susceptibility to neurodegeneration: the use of unbiased genetics to decipher critical regulatory pathways.. Neuroscience.

[91] Stadelmann C, Bruck W (2008). Interplay between mechanisms of damage and repair in multiple sclerosis,.. J, Neurol.

[92] Gongvatana A, Schweinsburg BC, Taylor MJ, Theilmann RJ, Letendre SL (2009). White matter tract injury and cognitive impairment in human immunodeficiency virus-infected individuals.. J Neurovirol.

[93] Barber SA, Herbst DS, Bullock BT, Gama L, Clements JE (2004). Innate immune responses and control of acute simian immunodeficiency virus replication in the central nervous system.. J Neurovirol.

[94] Sas AR, Bimonte-Nelson H, Smothers CT, Woodward J, Tyor WR (2009). Interferon-α causes neuronal dysfunction in encephalitis.. J Neurosci.

[95] Woods SP, Rippeth JD, Frol AB, Levy JK, Ryan E (2004). Interrater reliability of clinical ratings and neurocognitive diagnoses in HIV-1.. J Clin Exp Neuropsych.

[96] Report of a Working Group of the American Academy of Neurology AIDS Task Force (1991). Nomenclature and research case definitions for neurologic manifestations of human immunodeficiency virus-type 1 (HIV-1) infection.. Neurology.

[97] Reynolds NR, Sun J, Nagaraja HN, Gifford AL, Wu AW (2007). Optimizing measurement of self-reported adherence with the ACTG Adherence Questionnaire: a cross-protocol analysis.. J Acquir Immune Defic Syndr.

[98] Chowdhury IH, Chao W, Potash MJ, Sova P, Gendelman HE (1996). vif-negative human immunodeficiency virus type 1 persistently replicates in primary macrophages, producing attenuated progeny virus.. J Virol.

[99] Ou C-Y, Kwok S, Mitchell S, Mack D, Sninsky J (1988). DNA amplification for direct detection of HIV-1 in DNA of peripheral blood mononuclear cells.. Science.

[100] Hadas E, Borjabad A, Chao W, Saini M, Ichiyama K (2007). Testing antiretroviral drug efficacy in conventional mice infected with chimeric HIV-1.. AIDS.

[101] Sturn A, Quackenbush J, Trajanoski Z (2002). Genesis: cluster analysis of microarray data.. Bioinformatics.

[102] Hosack DA, Dennis G, Sherman BT, Lane HC, Lempicki RA (2003). Identifying biological themes within lists of genes with EASE.. Genome Biol.

[103] Wu HS, Murray J, Morgello S (2008). Segmentation of Brain Immunohistochemistry Images Using Clustering of Linear Centroids and Regional Shapes.. J Imaging Sci Technol.

