# Supplementary figures and images for: Significant Effects of Antiretroviral Therapy on Global Gene Expression in Brain Tissues of Patients with HIV-1-Associated Neurocognitive Disorders
Source: PLoS Pathog. 2011 Sep 1;7(9):e1002213. doi: 10.1371/journal.ppat.1002213 (PMC3164642; doi:10.1371/journal.ppat.1002213)

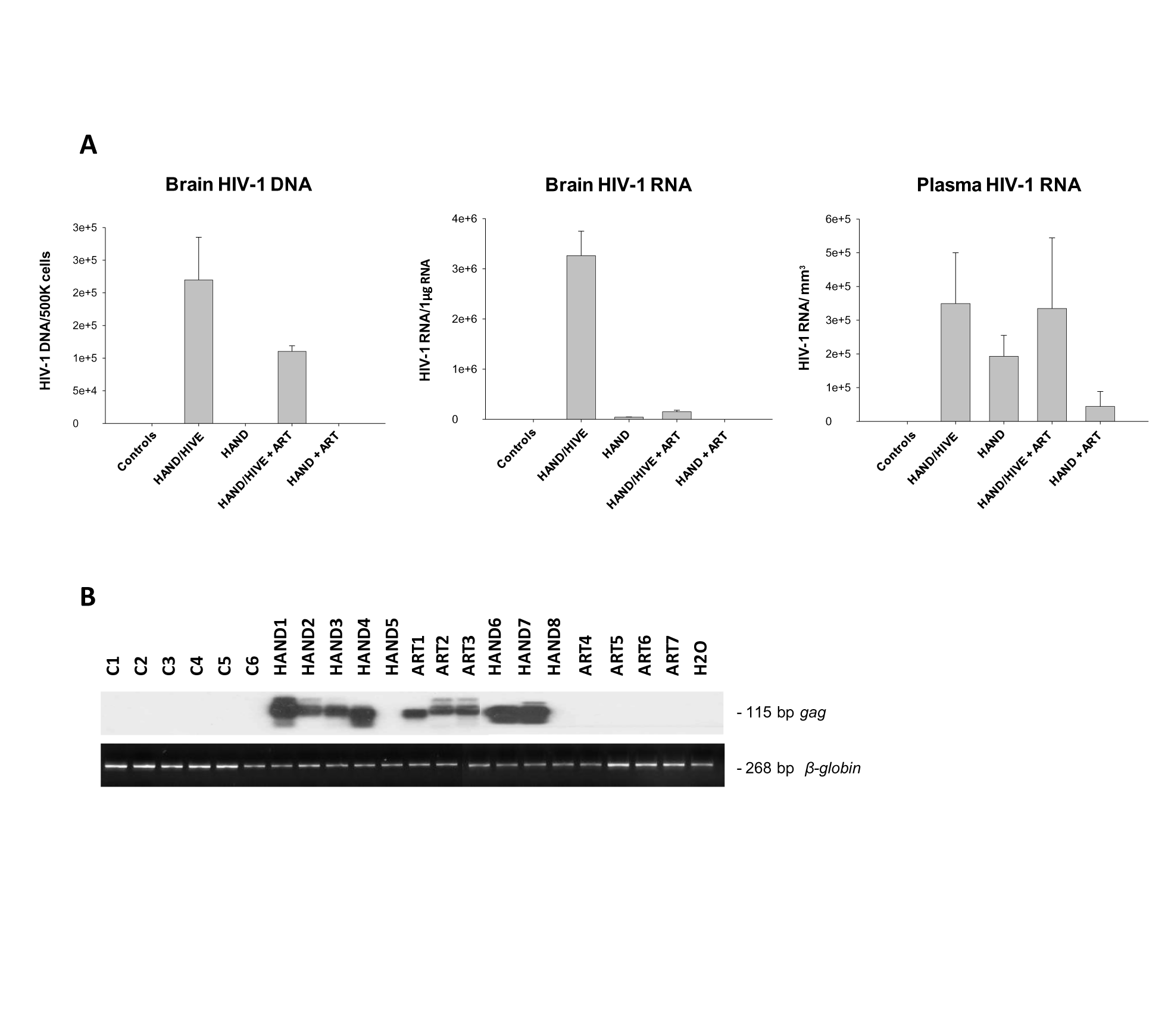

Supplement: Figure S1 — HIV-1 brain burdens of study subjects. A. Analysis of HIV-1 brain burdens in patient tissues by real-time PCR. Control: uninfected patients; HAND: patients with HAND without HIVE; HAND/HIVE: patients with HAND and HIVE; ART: treated patients of either category. HIV-1 DNA copies were prorated per 500,000 cells and HIV-1 RNA copies were per 1 µg RNA. HIV-1 plasma burdens are shown for comparison. B. Analysis of HIV-1 brain burdens by traditional PCR amplification and Southern blot hybridization. β-globin was used for normalization. For details, see Materials and Methods. (TIF) [file ppat.1002213.s001.tif]

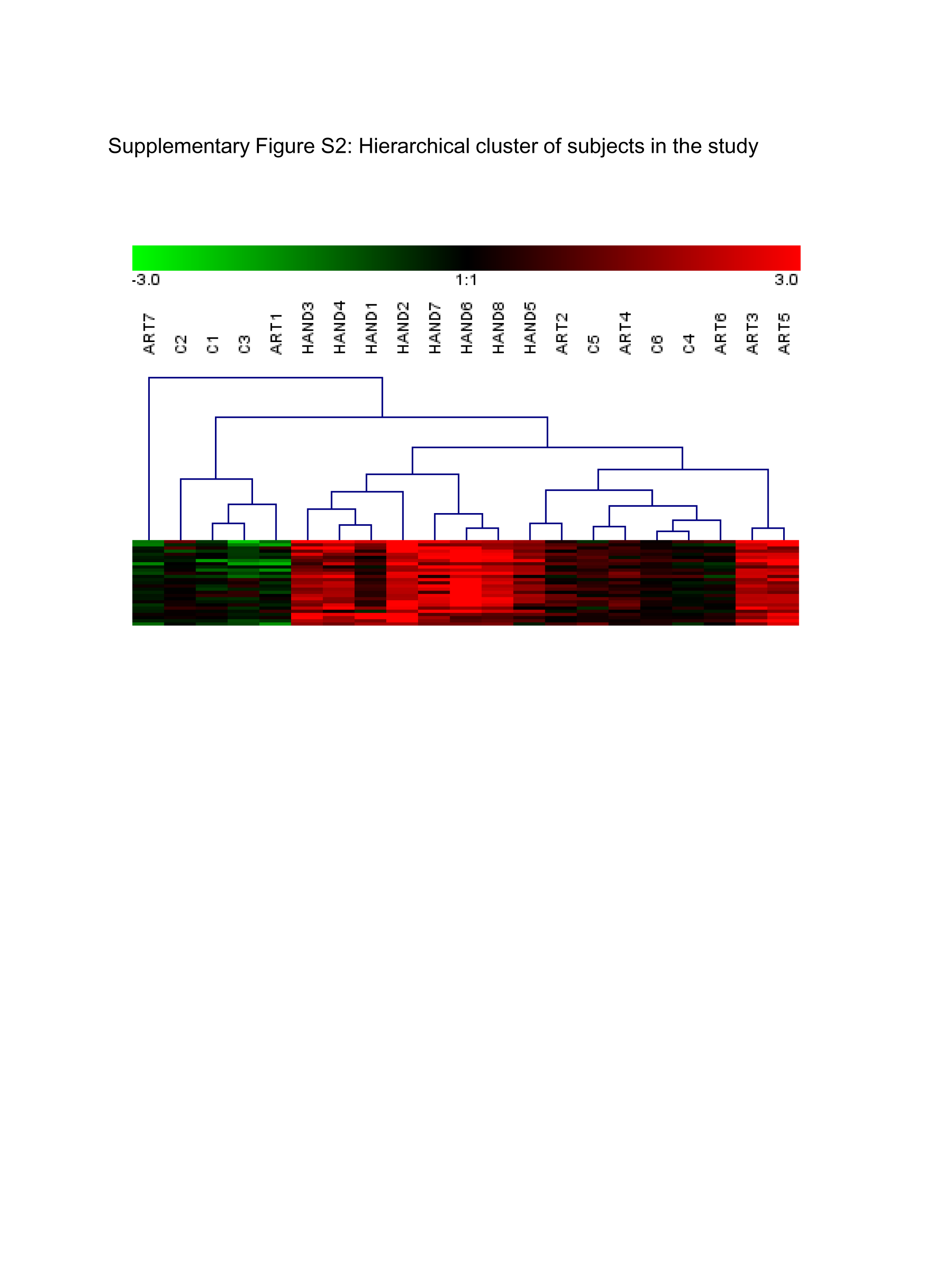

Supplement: Figure S2 — Hierarchical cluster analysis of study subjects. The Figure represents enlarged image of the cluster panel shown on the top of Figure 3A. The cluster tree shows computer-generated phenotypic relationship between the subjects in the study according to the presence and relative expression of 2073 HAND-associated transcripts listed in Supplementary Table S2. Note close phenotypic relationship between 7 out of 8 untreated HAND distinct from treated patients and controls. Treated patients clustered mostly together in two inter-related clusters with HIV-1-negative controls, (TIF) [file ppat.1002213.s002.tif]

## Slide 1
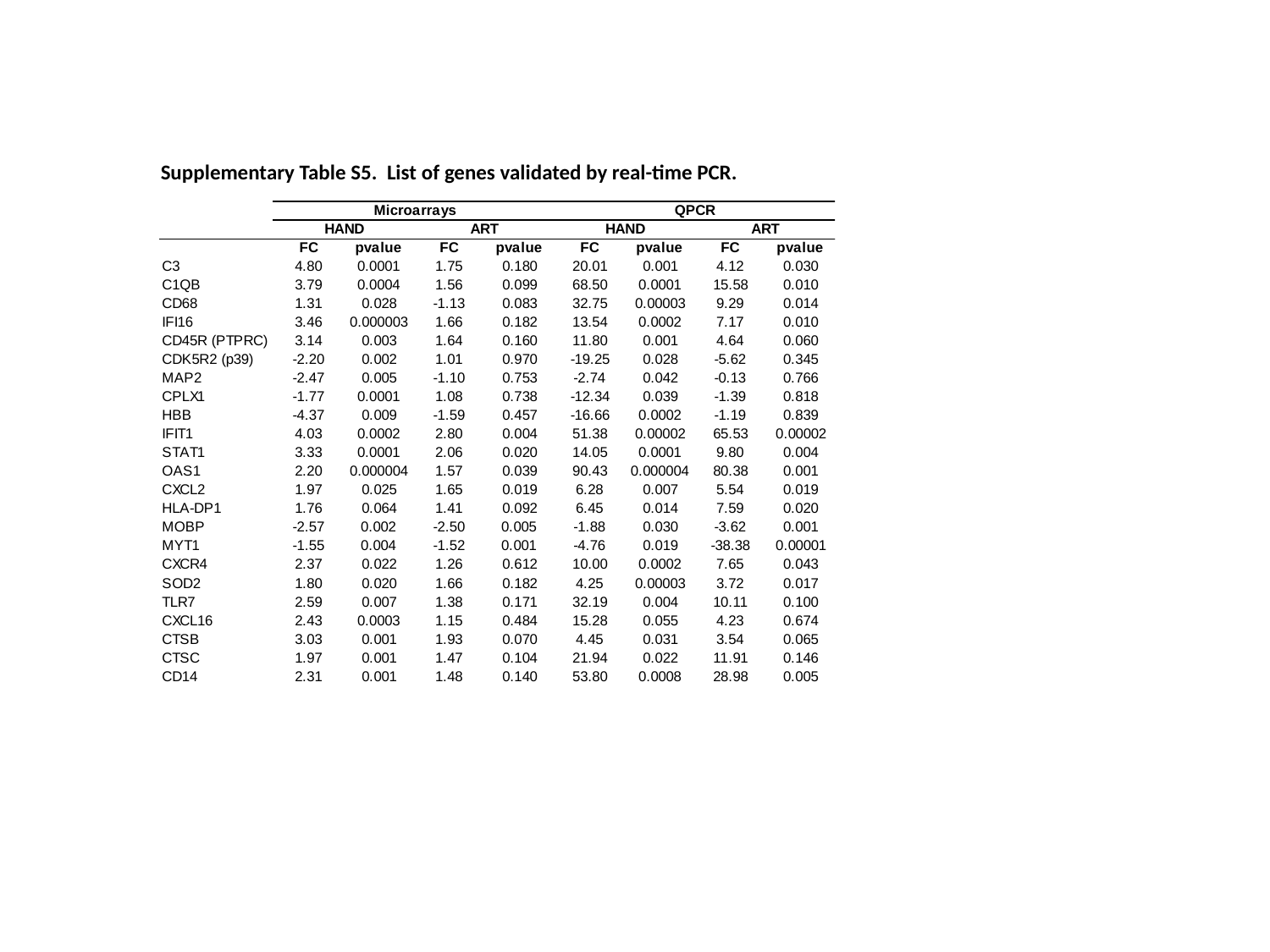

Supplementary Table S5. List of genes validated by real-time PCR.

Supplement: Table S5 — List of genes validated by real-time PCR. The Table shows average of 2 independent QPCR experiments for each gene listed; microarray results are shown for comparison. QPCR was conducted in duplicates on tissue samples adjoining to those used for microarray analysis; Taqman chemistry was employed as described in Materials and Methods. (PPT) [file ppat.1002213.s007.ppt]
