# Supplementary material for: Significant Effects of Antiretroviral Therapy on Global Gene Expression in Brain Tissues of Patients with HIV-1-Associated Neurocognitive Disorders
Source: PLoS Pathog. 2011 Sep 1;7(9):e1002213. doi: 10.1371/journal.ppat.1002213 (PMC3164642; doi:10.1371/journal.ppat.1002213)
Supplement: Table S9 — Gene expression commonalities with other array studies of brain tissues from patients with HAND. (PPTX) [file ppat.1002213.s011.pptx]

## Slide 1
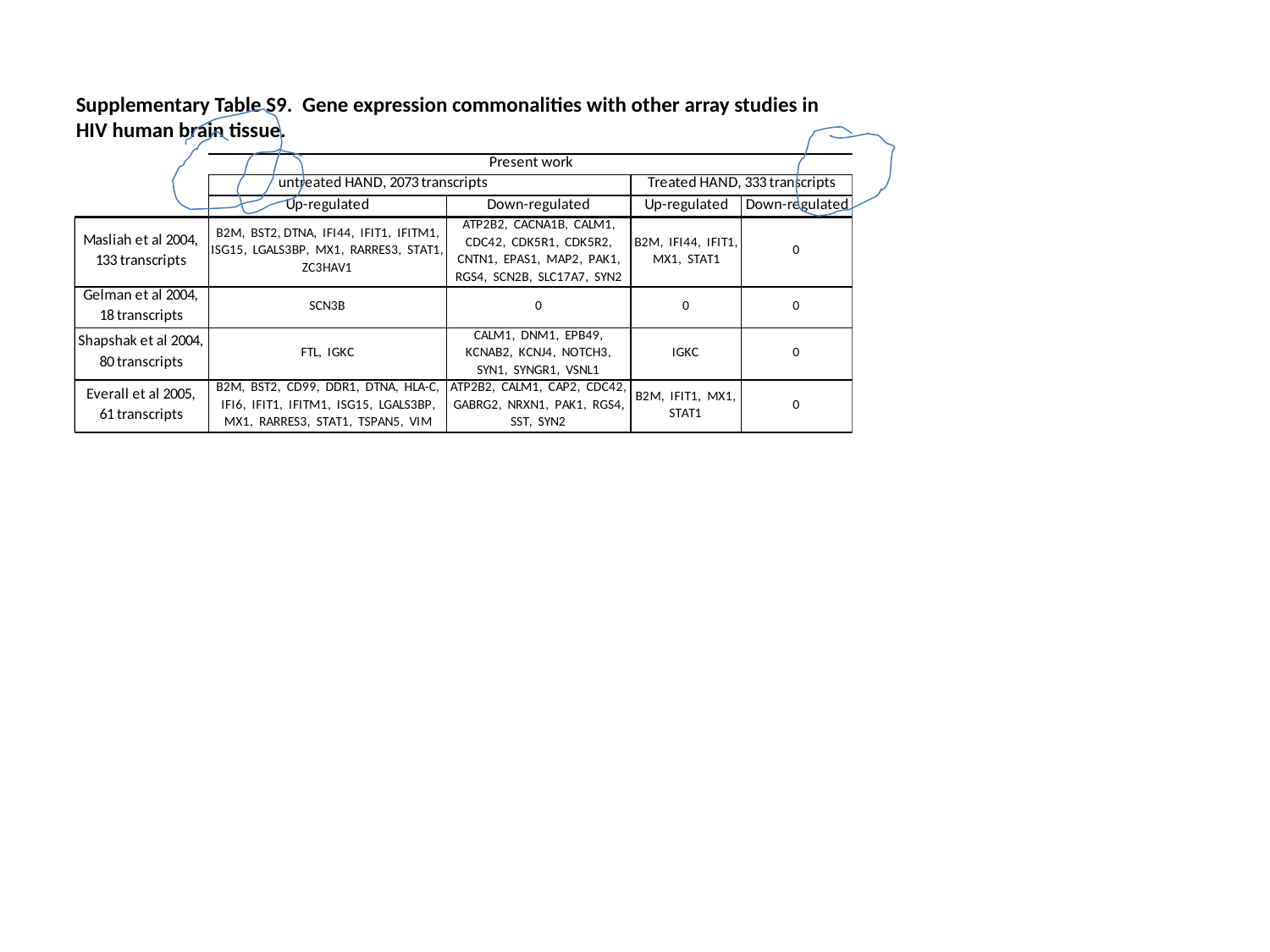

Supplementary Table S9. Gene expression commonalities with other array studies in HIV human brain tissue.
